# Engagement and Intersectionality in Digital Self-Management Interventions for Asthma and Chronic Obstructive Pulmonary Disease: Scoping Review

**DOI:** 10.2196/73431

**Published:** 2026-07-23

**Authors:** Martin Ruddock, Lucy Yardley, Katherine Bradbury, Tom Wilkinson, Ben Ainsworth

**Affiliations:** 1 School of Psychology, Faculty of Environmental and Life Sciences, University of Southampton, Building 44, Shackleton, University Road, Southampton, England, SO17 1BJ, United Kingdom, 44 02381208923; 2 University Hospital Southampton NHS Foundation Trust, NIHR Southampton Biomedical Research Centre, Southampton, England, United Kingdom; 3 School of Psychological Science, University of Bristol, Bristol, United Kingdom; 4 University of Bristol, The NIHR Health Protection Research Unit, Bristol, England, United Kingdom; 5 Clinical and Experimental Sciences, Faculty of Medicine, University of Southampton, Southampton, United Kingdom

**Keywords:** respiratory, asthma, chronic obstructive pulmonary disease, COPD, digital health interventions, self-management, health inequalities, intersectionality

## Abstract

**Background:**

Asthma and COPD are long-term respiratory conditions that require active self-management to improve quality of life and reduce health care burdens. Digital health interventions (DHIs) are increasingly used to support behavior change, symptom monitoring, and medication adherence, offering new opportunities for personalized care and real-time feedback. Understanding patient engagement with digital tools is essential for optimizing intervention design, improving clinical outcomes, and addressing potential inequalities in access and effectiveness. This review is informed by a novel conceptual foundation combining the Analyzing and Measuring Usage and Engagement Data (AMUsED) framework (for the analysis of digital engagement) and layered vulnerabilities (an intersectional approach). Together, these frameworks enable a more nuanced examination of how engagement is shaped by user behavior and structural factors.

**Objective:**

This study aims to evaluate how diverse patient groups engage with digital self-management interventions for asthma and COPD by examining the reporting of demographic characteristics, outcome measures, and usage data. The review also explores how these data types are combined in analysis, how authors interpret results, and the extent to which current reporting practices support equitable and meaningful evaluation of digital interventions.

**Methods:**

A 2-phase study selection process was applied. First, empirical studies were systematically identified if they reported demographic characteristics, clinical outcome measures, and usage data. Second, reported usage measures were reviewed to identify measures that were meaningful across interventions, defined as numerically comparable measures without subjective user input. Descriptive thematic analysis was conducted to map key concepts across studies, and patient and public involvement sessions were used to contextualize findings and inform interpretation of the results.

**Results:**

Twenty-seven studies met the inclusion criteria. Four comparable usage measures were identified, with studies reporting a mean of 2.15 (SD 0.74) usage measures. Thirteen (48.1%) studies reported 2 or more types of outcome measures (disease-specific self-reported, physiological, or other self-reported). Age, sex, and disease severity were reported in all studies, but characteristics linked to health inequalities were underreported; for example, 8 (29.6%) studies reported ethnicity and 2 (7.4%) reported socioeconomic status. Seventeen (62.9%) studies did not combine demographic, outcome, and usage data in analysis. Thematic analysis identified three cross-cutting issues: (1) limited characterization of engagement patterns, (2) dominance of single-trait demographic analysis, and (3) inconsistent conceptualization of health care support.

**Conclusions:**

This review is the first to integrate the AMUsED framework with layered vulnerabilities and to map how demographic, outcome, and usage data are reported and combined in digital self-management research. By identifying structural gaps in reporting and analysis, the review provides recommendations for more equitable and analytically rigorous digital health research. Strengthening reporting practices, particularly through richer usage data and intersectional analyses, will support clinicians, developers, and policymakers in tailoring digital self-management tools to diverse patient populations and improving real-world effectiveness.

## Introduction

### Asthma and Chronic Obstructive Pulmonary Disease

Asthma and chronic obstructive pulmonary disease (COPD) affect an estimated 8 million people in the United Kingdom [1]. The National Health Service (NHS) spends more than £4.9 billion (US $6.49 billion) treating these conditions annually [2]. Treatment costs and patient numbers are set to grow exponentially in the coming years [3]. Asthma and COPD are distinct conditions, but patients with these conditions experience similar symptoms, including chest discomfort, frequent coughing, and shortness of breath. Current clinical guidelines recommend self-management plans for both conditions [4,5]. The purpose of these treatment regimens is to ensure the best possible quality of life while minimizing the risk of exacerbations [6].

Inhaled medications are recognized to improve quality of life in both asthma and COPD. However, evidence suggests that inhaler adherence is below 50% for both conditions [7,8]. This poor adherence can lead to worsening quality of life and greater burdens on health care systems [9]. Consequently, self-management interventions offer a strategy to provide tailored behavior training, education, support for medication adherence, and symptom monitoring. Self-management interventions are integral to [10] supporting patients with long-term conditions, such as asthma and COPD, to develop an ability to balance lifestyle choices with risk of exacerbations [11]. Improvements can be observed in self-reported quality of life [12,13], reduced emergency visits, and reduced hospital readmissions [14,15].

Adherence to asthma and COPD self-management interventions remains problematic. However, digital platforms provide the potential for professionals to precisely monitor usage and understand how interventions might improve outcomes. For patients, digital interventions can help patients monitor their condition, promote correct medication use, and provide environmental alerts [16,17]. The evidence base for the effectiveness of digital interventions is still at an early stage and needs to demonstrate both improvements in individual health outcomes and infrastructure benefits [18].

### Patterns of Engagement: Gateway to Understanding Effectiveness and Tailoring

Digital health interventions are highlighted by the World Health Organization (WHO) [19] as a mechanism to improve quality, coverage, and equity of health care for all. A total of 96% of the UK population has internet access [20], and approximately 50% use that access for health information [21]. Digital interventions for the management of asthma and COPD have grown in popularity over the past fifteen years [22]. Digitalization has promised much, including supporting structural cost-effectiveness [23,24] and individual personalization [25]. Digital platforms can enable real-time reporting between clinicians and patients while also allowing platform-wide alerts and updates. However, evidence on the effectiveness of digital interventions for asthma and COPD remains unclear.

Systematic reviews and meta-analyses have reported small benefits that are not clearly sustained over longer time periods. Two such reviews each identified 3 studies of questionable quality that produced small and negative effects [26,27]. Meanwhile, narrative reviews suggest frameworks are developed to rigorously assess effectiveness and standardize reporting [28-30]. These reviews also suggest that reporting of different outcome measures makes it difficult to compare interventions. Conceptual models revolve around evidencing aspects of engagement and potential impacts on relevant outcome measures.

Engagement is the process of user investment through interaction with a digital platform [31]. It is dependent on both intervention provision (how and what resources are provided) and users (ability, accessibility, and comprehension), each contributing to engagement itself and what usage data are recorded. Engagement is not restricted to usage, but usage metrics provide a fixed record that can ostensibly be treated as neutral and impartial. Usage has therefore been conceptualized as “objective engagement” [32,33] that enables analysis of engagement and the potential impacts of such engagement. Examples of objective engagement include amount (eg, frequency and duration), breadth (eg, coverage of different aspects of a health condition), and depth (eg, level of detail) of user investment [32,33].

Identifying patterns in usage metadata is foundational to understanding engagement within and between interventions. These patterns offer a rigorous assessment beyond conventional analysis. Rather than comparing nonusers (controls) and users (interventional), another layer is added between nonusers and different types of users (eg, high- and low-intensity users). This shift recognizes the point that while usage is integral to digital interventions, it is not the ultimate aim. Intervention usage is a mechanism to support engagement with target behaviors, which leads to improved health outcomes [34]. Individuals may respond with lower or higher rates of engagement and/or after accessing specific content during critical periods (eg, after an exacerbation). The Analyzing and Measuring Usage and Engagement Data (AMUsED) framework [35] provides a methodology to develop greater understanding of engagement.

Identifying meaningful outcome measures is also essential to assessing effectiveness and engagement. Just as more usage does not necessarily indicate an improved outcome, different outcomes may be influenced by different usage. Furthermore, each user may expect different outcomes from the same intervention. For example, more physically able users may not require exercise features of an intervention but may benefit from personalized alerts. The concept of “effective engagement” attempts to identify patterns of engagement that correlate with a particular outcome, typically represented as behavior change or improved health outcomes [34]. Usage is analyzed in combination with the outcome, identifying optimal patterns within specific interventions. Effective engagement attempts to identify a specific amount of time, specific modules, or a critical event that statistically signals advancement toward a particular outcome. This provides conceptual scaffolding for more rigorous analyses of individual interventions and comparisons between interventions. The concept is demonstrated by Duckworth et al [36], who identified that user reports of increased reliever medications pre-empted reports of a decline in health. These data were used to suggest that digital interventions could prompt users to review and report their health when medication increases are detected [36], for example, that users could be encouraged to recognize and respond to a decline in health.

### Disease-Specific Health Disparities

Health disparities are differences in diagnosis, treatment, and outcome among patient subpopulations that are avoidable, unnecessary, and unjust [37]. These disparities can be the result of systematic, historical, and social injustices [38-41], including ageism, racism, and sexism. Disparities describe risks to patients with specific demographic characteristics, including experiencing symptoms at younger ages and with greater severity [42-46]. Structurally, health care services are used at increased rates, placing greater burdens on health care providers [47,48]. An example within asthma and COPD is the adjustment for “race” in spirometry readings, which sets lower expectations for lung health on the basis of crude categorizations of ethnicity, potentially leading to systematic underdiagnosis and undertreatment [49-52]. To understand how disparities manifest on digital platforms, it is necessary to recognize existing, disease-specific health disparities. Such recognition serves to better monitor how disparities may alter and/or reproduce in digital interventions [39]. For example, results of meta-analyses examining chronic conditions, including asthma and COPD, showed that minoritized ethnic populations benefited from digital interventions, while older adults and women did not [53].

Research on digital health interventions also needs to account for a digital divide. The concept of digital divide describes disparities in access to and engagement with digital platforms [54]. The Office for National Statistics [21] suggests minoritized ethnic groups, older adult groups, and women as more likely to lack internet access. Populations without internet access overlap those vulnerable to asthma and COPD inequalities. However, this does not necessarily mean disparities will be similarly mirrored among those able to engage with digital health interventions. A systematic review investigating engagement with patient-facing digital technologies identified no correlations among a narrow field of demographic characteristics [55]. A real-world study concluded that age, geographical location, and wealth were not barriers to using a digital COPD intervention [56]. It is currently unclear how disparities manifest on digital platforms [29,57-59]. While some suggest needs and resources will drive engagement [55], evidence is required by disease and population.

To summarize, it is important to identify and track subpopulations that are at risk of disease-specific disparities. However, researchers should be encouraged to add complexity and consider more intersectional approaches [60]. Intersectionality is the idea that a person or people are more complex than any single demographic characteristic, and research should account for multiple characteristics. Comprehensive, good-quality data are crucial to achieving this goal. Such data would enable policymakers and digital developers to identify specific vulnerabilities across heterogeneous populations and respond with more tailored strategies [61].

## Methods

### Aims and Objectives

This scoping review aims to explore gaps in knowledge regarding engagement with digital interventions for the self-management of asthma and COPD. This includes a detailed consideration of heterogeneous disease subpopulations for a more comprehensive understanding. To accomplish this, the following objectives were set:

1. To identify and categorize usage measures that studies report.
2. To identify and categorize physiological and self-reported outcome measures that studies report.
3. To identify and categorize the demographic characteristics that studies report.
4. To explore the number of studies that report analysis combining either demographic characteristics, outcome measures, or usage measures.
5. To describe how potential gaps in reporting are discussed in studies.

### Protocol and Registration

A scoping review methodology was selected because the objective was to describe and categorize the data that studies report and analyze rather than to evaluate intervention effectiveness. This scoping review was not registered. The search strategy followed PRISMA-S (Preferred Reporting Items for Systematic Reviews and Meta-Analyses Literature Search Extension) guidance [62] (see Checklist 1).

### Eligibility Criteria

To summarize the Population, Concept, and Context (PCC) framework (Table 1) [63], empirical studies were included based on four criteria: (1) participants had a diagnosis of asthma or COPD; (2) the intervention supported self-management via a digital platform and reported usage data; (3) a clinically validated outcome measure was reported; and (4) the study reported any demographic characteristics.

**Table 1.** Population, Concept, and Context framework.

PCC^a^ element	Definition
Population	Individuals with asthma or chronic obstructive pulmonary disease
Concept	The use of digital interventions for self-management (eg, education and training, exercise, monitoring, and reporting).
Context	Empirical research publications that report clinical outcome measures, participant demographics, and intervention usage measures.

a PCC; Population, Concept, and Context.

### Information Sources

Five academic databases were used, selected based on their relevance to the subject areas and pilot searches. The systematic search was originally conducted in February 2023, after consultation with a librarian, and updated in April 2026. No additional filters were applied (eg, humans, age groups, study design, or publication status, date, language, or publication type). Please see Multimedia Appendix 1 for further details on the search strategy.

### Selection of Sources of Evidence

The selection of databases and search terms was agreed upon by all authors after being reviewed by a senior librarian at the University of Southampton. The selection was based on authority, efficiency, relevance, and the ability to control the search. This ensured we captured relevant medical and technological journals. Search strategies combined controlled vocabulary (eg, MeSH) with free-text keywords tailored to each database.

One author (MR) and one reviewer completed title and abstract screening. Discrepancies were discussed and resolved remotely with a third reviewer. Full-text screening was conducted by one author (MR), and one reviewer screened 59.5% of records. During full-text screening, articles were reviewed and excluded in 2 phases using criteria developed by all authors. The 2-phase design reflects an approach used by Nouri et al [55] and was systematized by adhering to the AMUsED framework [35]. Phase 1 identified empirical research that reported all 3 types of data (demographic characteristics, clinical outcome measures, and usage). During phase 2, the research team identified and reviewed the range of reported usage measures; 4 comparable measures were selected based on their clinical relevance and objectivity (ie, numerical data without subjective user input).

Supplementary searches of ClinicalTrials.gov and International Standard Randomized Controlled Trial Number (ISRCTN) were undertaken to identify completed studies of digital self-management interventions for asthma and COPD. ClinicalTrials.gov was searched using structured fields (condition: “asthma,” “Chronic Obstructive Pulmonary Disease,” and “COPD”; other terms: “digital,” “mobile,” “smartphone,” “app,” “web,” “telehealth,” “telemedicine,” and “self-management”). ISRCTN was searched using paired keyword combinations of respiratory condition terms with digital-intervention terms due to platform constraints. ISRCTN does not support Boolean nesting or field-specific searching; therefore, paired keyword combinations were required to ensure comprehensive retrieval. Both registries were restricted to completed studies, with no date limits applied. Titles and summaries were screened for relevance (see Multimedia Appendix 1 for full details). Only completed studies were included because the review required empirical reporting of usage, demographic characteristics, and validated outcome measures, which are not available for ongoing trials.

To confirm, online resources or websites, handsearching, citation chasing, and author contact were not undertaken, as these were not part of the planned search methodology for this scoping review. Finally, truncation and wildcard operators (eg, *) were used where appropriate (see Checklist 1).

### Data Charting Process

All data extraction processes were discussed among all authors, with regular updates. Descriptive statistical data were extracted and reviewed first by MR. Table 2 provides a list of data items for extraction. Data for descriptive thematic analysis were extracted by MR; line-by-line coding was performed in NVivo (Lumivero LLC). Descriptive themes were developed in NVivo and through thematic mapping (see Multimedia Appendix 2 for an overview of the process). This thematic mapping aligns with Joanna Briggs Institute (JBI) guidance [63] for collating and summarizing scoping review results.

**Table 2.** Data items.

Data item	Definition
Demographic characteristics reported	Each characteristic was listed individually. The total number of characteristics reported.
Outcome measures reported	Each outcome measure was listed individually. Outcome measures were categorized into 3 types (condition-specific self-reported, physiological, and other self-reported). A count of each category.
Usage measures reported	Each usage measure was listed individually. Meaningful measures were selected for focus.
Types of analysis reported	Each analysis was listed individually. Analysis was categorized into five types based on whether different data types were combined: (1) no combinations; (2) demographic and usage; (3) usage and outcome; (4) demographic and outcome; and (5) demographic, outcome, and usage.
Descriptive thematic analysis	All text after the Results section (eg, Discussion, Implications, Limitations, and Conclusions).

### Critical Appraisal of Individual Sources of Evidence

The National Institutes of Health (NIH) quality assessment tool [64] was used to appraise methodological rigor, focusing on aspects such as sample selection, measurement validity, and risk of bias. Although scoping reviews do not typically require quality appraisal, this additional step provides contextual insight into the methodological strengths and limitations of the included evidence base.

### Synthesis of Results

The results section primarily reports descriptive information, such as demographic characteristics, types of outcome measures, and types of usage data. These data respond to the objectives of the study, identifying current reporting practices in research on digital interventions for the management of asthma and COPD. Following JBI guidance for scoping reviews [63], we analyzed the evidence by mapping patterns across usage, outcomes, and demographic characteristics to identify consistencies, contradictions, and areas where evidence was absent. This enabled us to interpret how reporting practices shape the field and where conceptual or methodological gaps persist.

Following JBI guidance [63], we conducted a descriptive thematic analysis of extracted data to collate and map key concepts. Coding followed Thomas and Harden [65] for a structured approach, restricted to semantic, descriptive themes consistent with scoping review methodology. All text after the “Results” section was treated as data and imported into NVivo for analysis (ie, Discussion, Implications, Limitations, and Conclusion sections). After familiarization through multiple readings of the data, codes were categorized into initial descriptive codes; these codes were collated into broader conceptual descriptive themes. These descriptive themes are presented as a narrative in the Results. Co-authors reviewed the themes, including the conceptual descriptive themes generated.

Finally, patient and public involvement (PPI) sessions were organized to discuss themes that developed in the results and how to interpret them. This follows best practice in health research and encourages building community partnerships among populations [66-68]. The PPI participants were members of the Priory Road Group based in Hampshire, England. Members included individuals with a diagnosis of asthma or COPD, those with caregiving experience, or health care professionals. The purpose was to discuss themes, missing variables, and prioritization. To accomplish this, preset matrix scoring activities were used, a visual participatory tool that identifies and prioritizes a range of categories [69]. The preset categories were identified from the results; one activity discussed themes of engagement, and another activity discussed demographic characteristics (see Multimedia Appendix 3). Outcomes of PPI sessions were integrated into the Discussion section to provide context and real-world relevance.

## Results

### Overview of Included Studies

**Figure 1.**
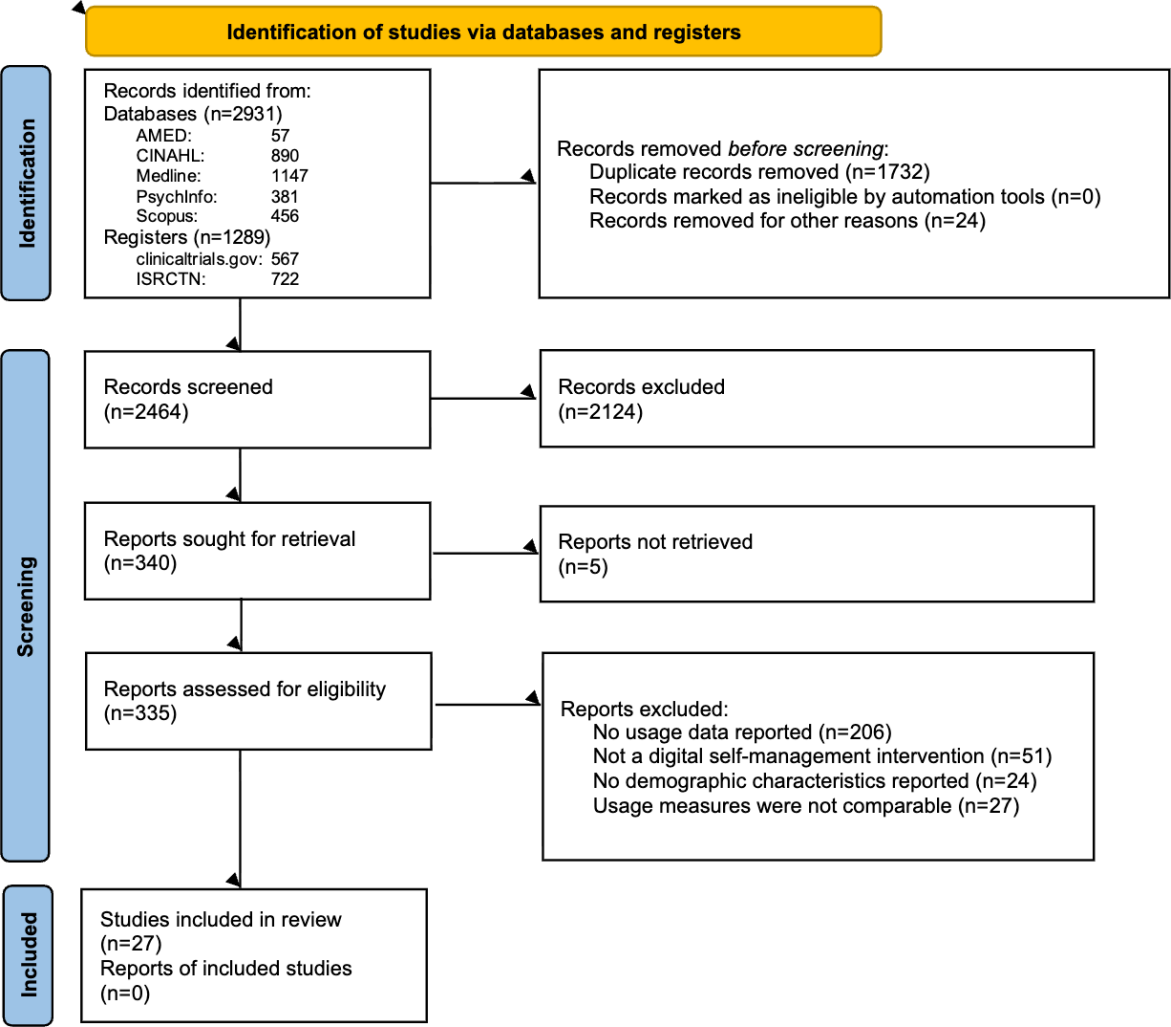
PRISMA (Preferred Reporting Items for Systematic Reviews and Meta-Analyses) 2020 flow diagram.

The evidence is reported in adherence to PRISMA-ScR (Preferred Reporting Items for Systematic Reviews and Meta-Analyses Extension for Scoping Reviews) guidelines [70] (see Checklist 2). The PRISMA (Preferred Reporting Items for Systematic Reviews and Meta-Analyses) 2020 flow diagram (Figure 1) shows that 2464 unique records were identified through searches of 5 academic databases and 2 registries, with 27 records included in the final analysis. Records were managed in EndNote during screening and deduplication. The software identified duplicates that were then verified by an author (MR). Additional duplicates were identified and checked by an author (MR) and reviewers. The 27 [13,25,56,71-94] included studies investigated 26 unique digital interventions (one intervention was evaluated in 2 separate studies). Eighteen [13,71-73,75-77,82-85,87-93] studies focus on asthma and 9 on COPD [25,56,74,78-81,86,94]. Table 3 provides an overview of study characteristics, focusing on extracted data. The NIH Quality Assessment tool [64] raised few concerns (Multimedia Appendix 4).

**Table 3.** Study characteristics.

Author	Design and duration	Country	Population	Intervention description	Outcome measures	Usage measures	Demographics reported (n)
Chan et al [71]	RCT^a^; 12 months	Hawaii, United States	6‐17 yrs; asthma: persistent	Web-based: (1) asthma education; (2) video recording of peak-flow/inhaler use forwarded to website; (3) daily asthma diaries; (4) 24/7 case-manager communication	ED^b^ visits; FEF^c^; FEV^d^; FVC^e^; hospitalizations; no asthma-specific measure	In-app time	4
Mammen et al [72]	Single-arm real-world mixed methods; 6 months	New York, United States	18‐44 yrs; asthma: persistent	App: (1) symptom monitoring; (2) nurse follow-up via Zoom (Zoom Communications, Inc); (3) guideline-based CDS^f^ calculating severity, control, and therapy	ACQ^g^; FEV; PFM^h^; AQLQ^i^	Logins; in-app time; module use	15
Lau et al [73]	RCT; 12 months	Australia	>18 yrs; asthma	Web-based: (1) evidence-based asthma info; (2) monthly email reminders; (3) interactive features (forum, poll, PHR)^j^	Written AAP^k^; no ACT^l^/ACQ/CARAT^m^	Frequency of access	5
Marklund et al [74]	RCT mixed methods pilot; 12 months	Sweden	Adults; COPD^n^	Web-based: (1) education; (2) strategies (exercise, breathing, observing symptoms, reducing exertion); (3) physical activity recording	FVC; FEV; CAT^o^; MRC^p^	Logins; in-app time	9
Talboom-Kamp et al [94]	RCT parallel cohort; 18 months	Netherlands	Adults; COPD	Web-based: (1) education; (2) goal-setting and monitoring; (3) clinician access for consultations	CCQ^q^	Logins; module use	4
Khusial et al [75]	RCT; 6 months	The Netherlands and the United Kingdom	>18 yrs; asthma	App: (1) diary/AAP; (2) personalized goals with clinician	ACT; mini-AQLQ	Module use	6
Kosse et al [76]	Cluster RCT; 6 months	The Netherlands	12‐18 yrs; asthma	App: (1) symptom monitor; (2) medication alerts; (3) educational/motivational videos; (4) peer chat; (5) pharmacist chat; (6) adherence questions	CARAT	Frequency; module use	3
Real et al [77]	RCT pilot; 4 months	Cincinnati, United States	4‐11 yrs; asthma	App: (1) didactic videos; (2) reinforcement games; (3) electronic AAP; (4) inhaler-type recognition via camera	C-ACT^r^	In-app time; module use	5
Velardo et al [25]	RCT mixed methods parallel; 12 months	Oxford, United Kingdom	>40 yrs; COPD	App: (1) diary (pulse, O₂ saturation); (2) clinician communication; (3) self-management feedback	SpO₂^s^; BPM^t^; no COPD-specific measure	Frequency; logins	3
Knox et al [78]	Real-world pilot; 6 weeks	Wales, United Kingdom	>40 yrs; COPD	App: not sufficiently described	UCOPD^u^; exacerbations; GP^v^/hospital attendance; steroid use	Frequency	4
Tabak et al [79]	RCT pilot; 9 months	Twente, the Netherlands	Adults; COPD	Web-based: (1) exercise program; (2) activity coach; (3) self-management module; (4) teleconsultation	CCQ; ED visits; LOS^w^; hospitalizations	Logins; in-app time; module use	6
Boer et al [80]	RCT; 12 months	Nijmegen, the Netherlands	>40 yrs; COPD	App: (1) personalized medication instruction; (2) breathing/coughing techniques; (3) energy distribution; (4) HCP^x^ contact; (5) “measure again tomorrow”	Exacerbation-free time; TEXAS^y^ system	Logins; frequency	7
North et al [81]	RCT feasibility; 3 months	England, United Kingdom	>45 yrs; COPD	App: (1) education; (2) 6-week online PR^z^; (3) inhaler videos; (4) environmental alerts	CAT; exacerbations; readmission; inhaler technique; PAM^aa^	Logins	4
Morita et al [82]	RCT; 12 months	Ontario, Canada	>18 yrs; asthma	App: (1) journaling symptoms/medication; (2) zone-of-control review; (3) action plans	ACT (baseline only)	Logins	5
Cooper et al [56]	Feasibility; 12 months	Scotland, United Kingdom	>40 yrs; COPD	App: (1) symptom scoring; (2) inhaler technique; (3) virtual PR	Health service usage	Logins; module use	5
Benfante et al [83]	Real-world pilot; 6 months	Palermo, Italy	>18 yrs; severe asthma	App: (1) daily symptom monitoring via VAS; treatment unchanged	VAS^ab^	Frequency	3
Ahmed et al [84]	RCT pilot; 9 months	Montreal, Canada	18‐69 yrs; asthma: poorly controlled	Web-based: (1) personal health info; (2) tailored education; (3) self-management feedback	MAQLQ^ac^; ACT; BMQ^ad^; PHQ-9^ae^; EQ-VAS^af^; ED/hospitalization	Logins; module use	5
Salim et al [85]	Real-world mixed methods; 3 months	Malaysia	>18 yrs; asthma	App: (1) education; (2) self-management; (3) behavior change; (4) social support	GINA^ag^; symptom control; severe attacks	Logins	7
Glynn et al [86]	RCT; 12 months	Ireland	>18 yrs; COPD	App: (1) education; (2) symptom tracking; (3) HCP communication; (4) goal setting; (5) motivational messages	Clinical attendance due to exacerbation	Logins	9
Gustafson et al [87]	RCT; 12 months	Wisconsin, United States	4‐12 yrs; asthma: poorly controlled	Web-based: (1) information; (2) adherence strategies; (3) decision tools; (4) support services	ACQ; symptom-free days	Frequency; logins; in-app time; modules	4
Genberg et al [88]	Real-world retrospective; 12 months	Helsinki, Finland	>18 yrs; asthma	App: (1) education; (2) self-management; (3) diary; (4) notifications; (5) messaging; (6) questionnaires	Clinical visits; medication use	Module use	8
Silverstein et al [89]	RCT secondary; 2 months	New York, United States	>18 yrs; asthma: persistent	App: asthma education; outcome data collection	PHQ-9; ACT; AQLQ; eHEALS^ah^; NVS^ai^	Average logins	4
van der Berg et al [90]	Pilot mixed methods; 12 months	Leiden, the Netherlands	>18 yrs; asthma	App: (1) SABA use; (2) symptoms; (3) education	CARAT	Frequency; in-app time; modules	5
Silberman et al [91]	RCT; 12 months	United States (nationwide)	18‐64 yrs; asthma	App: daily entries (symptoms, triggers, meds); smart nudges; AAP; wearable integration	ACT; unplanned care; adherence; WPAI^aj^	Symptom logs; app opens	7
Bruzzese et al [92]	RCT pilot; 4 months	New York City, United States	13‐18 yrs; asthma: uncontrolled	App: (1) info & feelings; (2) communication skills; (3) medication use; (4) self-management skills; (5) barriers; (6) triggers; (7) stress; (8) personalized feedback	ACT; PAQLQ^ak^	In-app time; modules	4
Newhouse et al [93]	RCT feasibility; 2 weeks	England, United Kingdom	>18 yrs; asthma: chronic	Web-based: educational content (early signs, symptoms, coping, HCP communication, emotions)	ACT	Logins; in-app time; modules	5
Greenwell et al [13]	RCT feasibility mixed methods; 12 months	England, United Kingdom	>18 yrs; asthma: mild but impaired	Web-based: (1) adherence; (2) service use; (3) breathing retraining; (4) stress management; (5) social support; (6) lifestyle	ACQ; AQLQ; FEV/FVC	Logins; in-app time; modules	10

a RCT: randomized controlled trial.

b ED: emergency department.

c FEF: forced expiratory flow.

d FEV: forced expiratory volume.

e FVC: forced vital capacity.

f CDS: clinical decision support.

g ACQ: asthma screening questionnaire.

h PFM: peak flow meter.

i AQLQ: asthma quality of life questionnaire.

j PHR: platelet-to-high-density lipoprotein cholesterol ratio.

k AAP: asthma action plan.

l ACT: Asthma Control Test.

m CARAT: Control of Allergic Rhinitis and Asthma Test.

n COPD: chronic obstructive pulmonary disease.

o CAT: COPD assessment test.

p MRC: Modified Medical Research Council Dyspnea Scale.

q CCQ: clinical COPD questionnaire.

r C-ACT: childhood asthma control test.

s SpO₂: peripheral capillary oxygen saturation.

t BPM: basic metabolic panel.

u UCOPD: unknown/undiagnosed COPD.

v GP: general practitioner.

w LOS: length of stay.

x HCP: health care professional.

y TEXAS: Telephonic Exacerbation Assessment System.

z PR: pulmonary rehabilitation.

aa PAM: patient activation measure.

ab VAS: visual analogue scale.

ac MAQLQ: Modified Asthma Quality of Life Questionnaire.

ad BMQ: Beliefs about Medicines Questionnaire.

ae PHQ-9: Patient Health Questionnaire-9.

af EQ-VAS: euroqol visual analogue scale.

ag GINA: global initiative for asthma.

ah eHEALS: ehealth Literacy Scale.

ai NVS: newest vital sign

aj WPAI: Work Productivity and Activity Impairment.

ak PAQLQ: Pediatric Asthma Quality of Life Questionnaire.

All studies [25,56,71-84,86-94] were conducted in the United States or Western Europe except for one study [85] from Malaysia (see Table 3). The studies contained a minimum of 15 and a maximum of 899 participants, with a total of 4019 participants (n=1421 control; n=2598 intervention). Twelve studies [25,71,73,75,76,80,82,86,87,89,91,94] were full RCTs, and 8 [13,74,77,79,81,84,92,93] were feasibility or pilot adaptations of an RCT model. Additionally, 6 [13,25,72,74,85,90] were mixed methods and 5 were real-world studies [72,78,83,85,88]. Nineteen interventions investigated mobile phone apps, and 8 were web-based interventions. This is largely reflected by the year of the publication; the 6 studies [71,73,79,84,87,93] published before 2017 were all web-based.

### Usage Measures (Objective 1)

**Figure 2.**
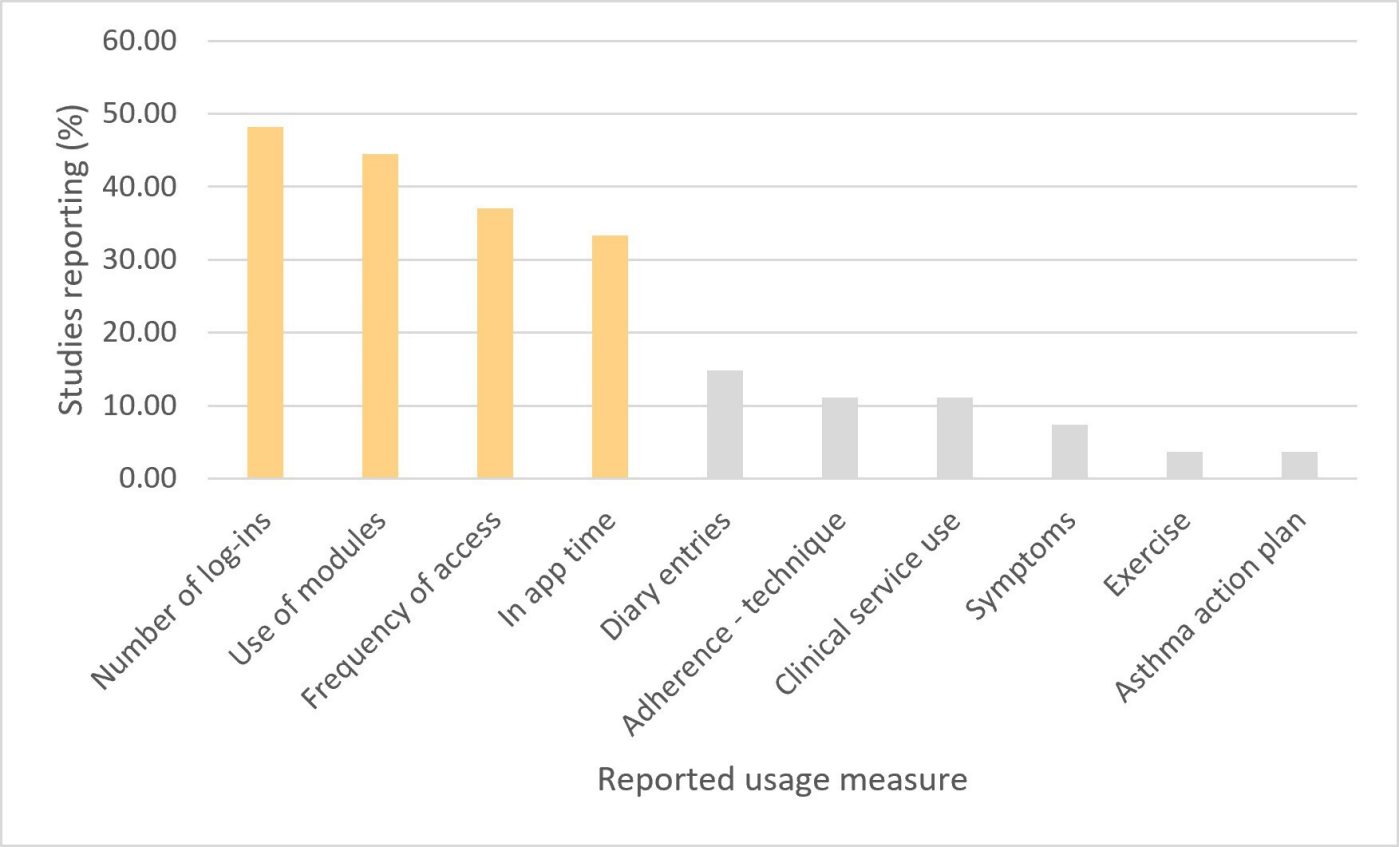
Reported usage measures (comparable measures in yellow).

Full-text screening identified 284 studies that investigated relevant digital health interventions; the majority of these did not report any usage measures (n=206, 72.5%). Four comparable usage measures were identified by adhering to the AMUsED framework [35]: (1) frequency of access, (2) in-app time, (3) number of logins, and/or (4) studies that reported the use of modules (ie, usage of any specific components such as exercise or inhaler technique). Studies that did not report any of these 4 measures were excluded (n=27). An average of 1.63 comparable usage measures were reported by the included studies, with the most frequently reported measure being the number of logins (Figure 2). Most studies reported at least 2 usage measures (mean 2.15, SD 0.74). In addition to the 4 comparable measures, 9 studies [25,56,71,73,75,79,82,91,93] reported at least one other usage measure.

### Outcome Measures (Objective 2)

Outcome measures were categorized into three groups to standardize the diversity of validated instruments: (1) condition-specific self-reported measures (eg, Childhood Asthma Control Test [C-ACT] and Asthma Control Questionnaire [ACQ]); (2) clinical and physiological measures (eg, forced expiratory volume [FEV] and oxygen saturation); and (3) other self-reported measures (eg, patient activation measure and wider quality of life measures). Each of these categories contains directly comparable instruments with a full description listed in Table 3.

As outlined in Table 4, 14 (51.9%) studies [25,56,73,75-77,82,83,86,88,90,92-94] reported one type of outcome measure (9 disease-specific self-reported and 5 physiological). Almost half the studies [13,71,72,74,78-81,84,85,87,89,91] (48.1%) reported at least 2 types of outcome measure. Four studies [74,81,84,91] reported all 3 types of outcome measure. Twenty studies [13,72-79,81,82,84,85,87,89-94] (74.1%) reported a disease-specific outcome measure, with one study [82] reporting at baseline only.

**Table 4.** Number of types of outcome measures reported.

Types of outcome measures	One reported	Two reported	Three reported
Condition-specific self-reported, n (%)	9 (33.33)	7 (25.93)	4 (14.81)
Physiological, n (%)	5 (18.52)	7 (25.93)	4 (14.81)
Other self-reported, n (%)	0 (0.00)	4 (14.81)	4 (14.81)
Total studies, n (%)	14 (51.85)	9 (33.33)	4 (14.81)

### Demographic Characteristics (Objective 3)

**Figure 3.**
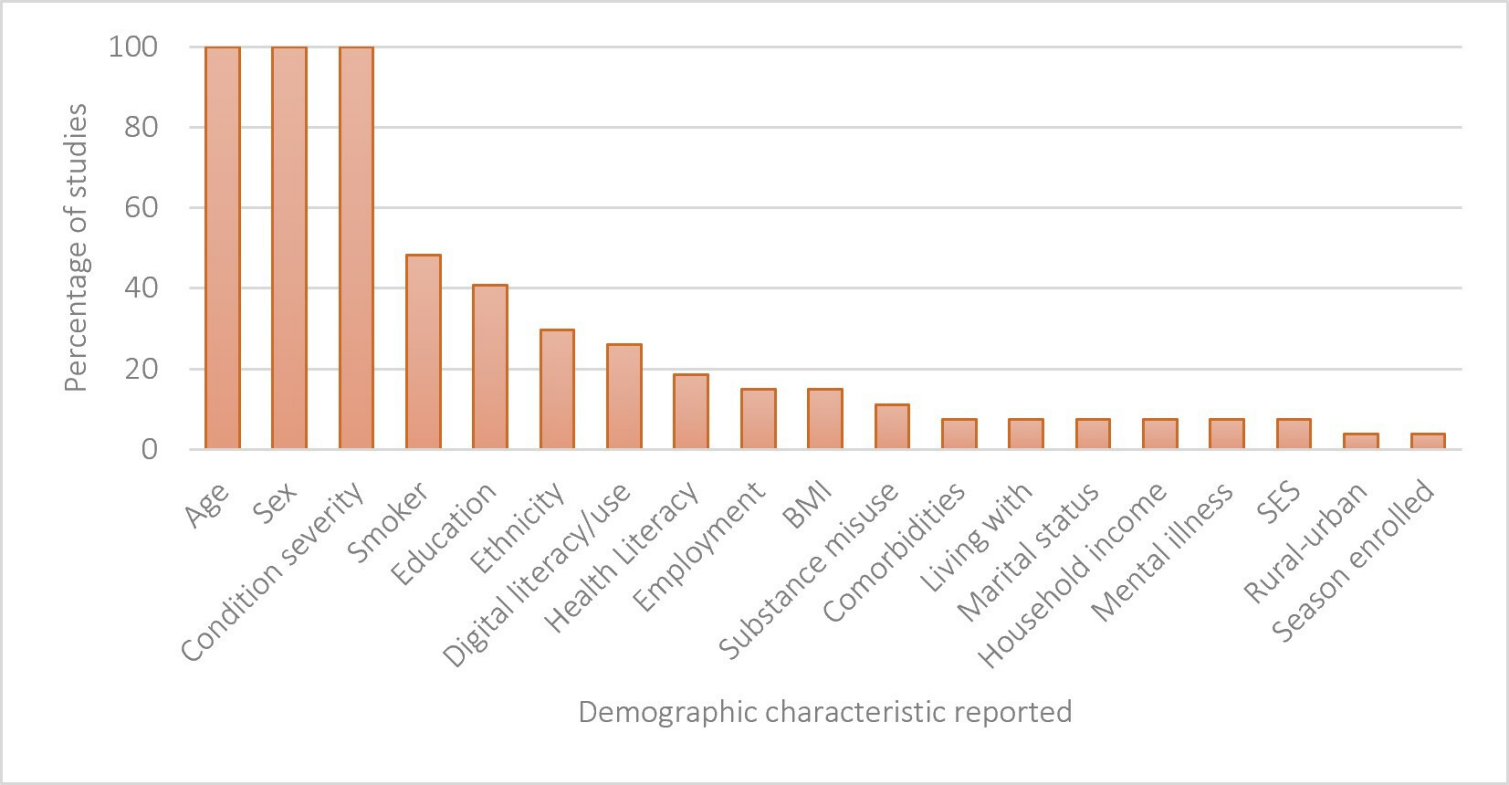
Demographic characteristics reported in studies. SES: socioeconomic status.

As displayed in Figure 3, all studies reported data on 3 characteristics, including age, disease severity, and sex. Comorbidities [72,80] and health literacy [71,74,77,89] were reported in less than 20% of studies. A total of 2587 out of 4022 (66.7%) participants were identified as female. Sex distribution was not explicitly reported for the control groups in 2 studies [25,94]. Thirteen studies (48.1%) reported either a measure of socioeconomic status (SES) or a proxy of SES (ie, education, employment, or income). Ethnicity was reported in 8 studies [13,72,77,85,87,89,92,93] (29.6%), although this was dichotomous in 3 studies [13,77,87] with participants being described as either African American or not, Black or non-Black, and White or other. Across all studies, a total of 705 out of 4022 participants (17.5%) could be identified as belonging to an ethnically underrepresented group. This was concentrated in 3 studies [87,89,91] that accounted for 81.8% (n=577) of such participants. There was a lack of consistency in reporting many characteristics, with most being reported no more than twice.

### Types of Data Combined in Analysis (Objective 4)

**Figure 4.**
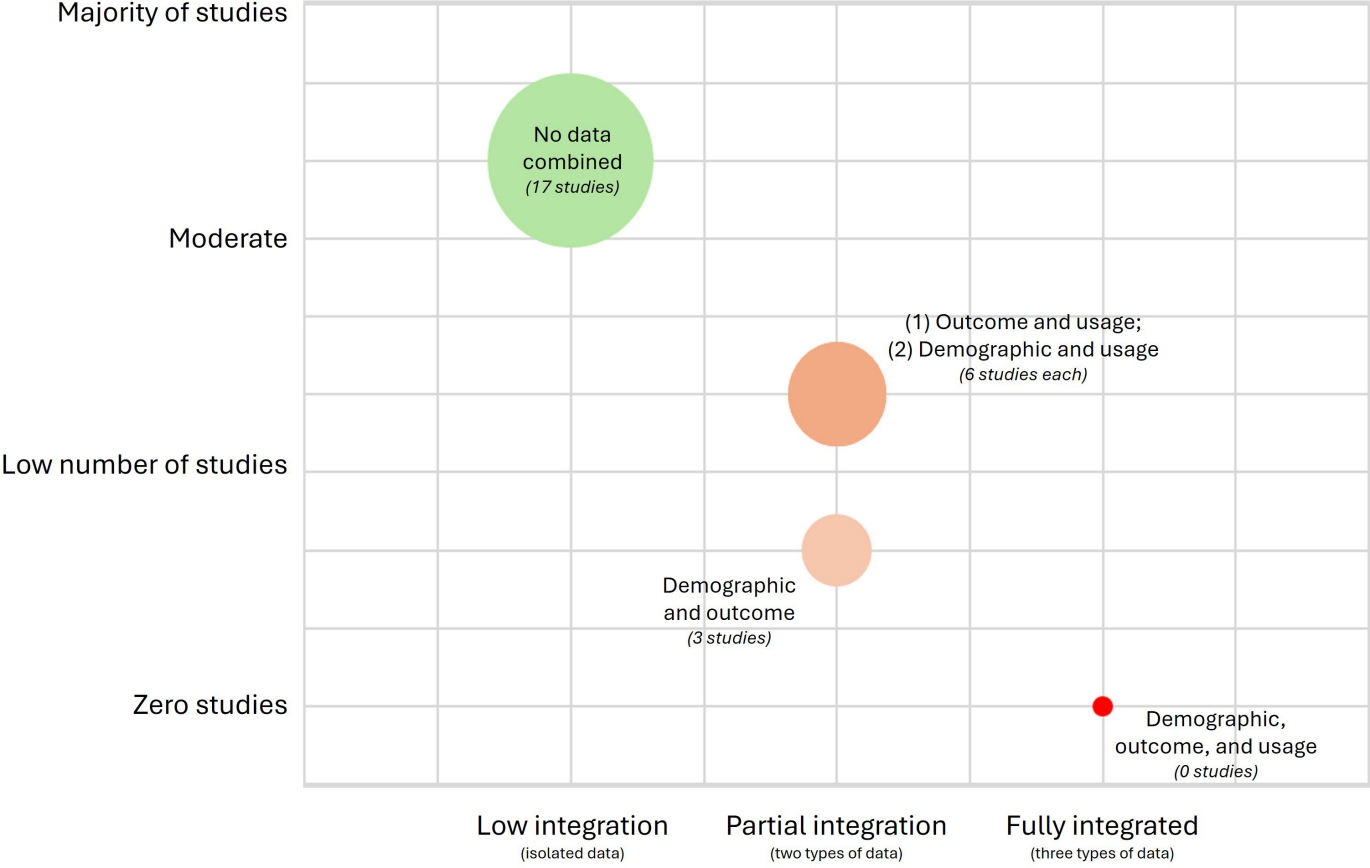
Evidence gap map: types of analysis studies conducted.

A total of 10 (37.0%) studies [72,74,76,77,82,83,88,89,91,92] combined different types of data in analysis (demographic, outcome, and usage). One study reported only statistically significant findings. These results are displayed in Figure 4.

Usage and outcome measurements were combined in 6 studies [76,77,83,88,89,91] (22.2%). One categorized usage into levels of usage (eg, high- and low-intensity users) but did not find any significant improvements, although low-intensity users were more likely to complete outcome measures. Three studies [76,77,88] suggested that more usage improved asthma outcomes. Additionally, one study [88] indicated a greater reduction in exacerbations compared to controls but not a greater reduction in health care visits.

Demographic characteristics and usage measures were combined in 6 studies [74,76,82,89,91,92] (22.2%). Significant differences were associated with usage and age (n=1) [82], ethnicity (n=1) [91], digital literacy (n=1) [89], and sex (n=2) [76,92]. Those aged 50 years and older were associated with increased usage. One study [91] reported that usage rates among African Americans were lower compared to other ethnic groups. One study [89] suggested that higher digital literacy correlated with greater usage. Two studies [76,92] reported higher usage among female participants who were more likely to complete interventions, using them more often and for longer periods. One study [89] also noted that low- or nonusage was associated with poor health literacy. Another study reported increased usage when a physician administered the intervention.

Three studies [72,89,91] (11.1%) analyzed demographic characteristics and outcome measures in combination. One [72] suggested improved breathing capacity for those with a range of characteristics, including smokers (vs nonsmokers), males (vs females), and those educated to a high school level (vs college level and above). Additionally, patients with worse asthma control improved their symptoms the most. This study also confirmed no significant differences to ACQ according to ethnicity, comorbidities, education, sex, and smoking. These results were supported by a second study [89] that found no significant differences in outcome and demographics (age, education, ethnicity, and sex). Finally, one study [91] suggested that outcome was moderated by race, but this was caveated by lower engagement among that group.

No studies performed a combined analysis of all 3 data types (demographic, outcome, and usage).

### Results of Descriptive Thematic Analysis (Objective 5)

**Figure 5.**
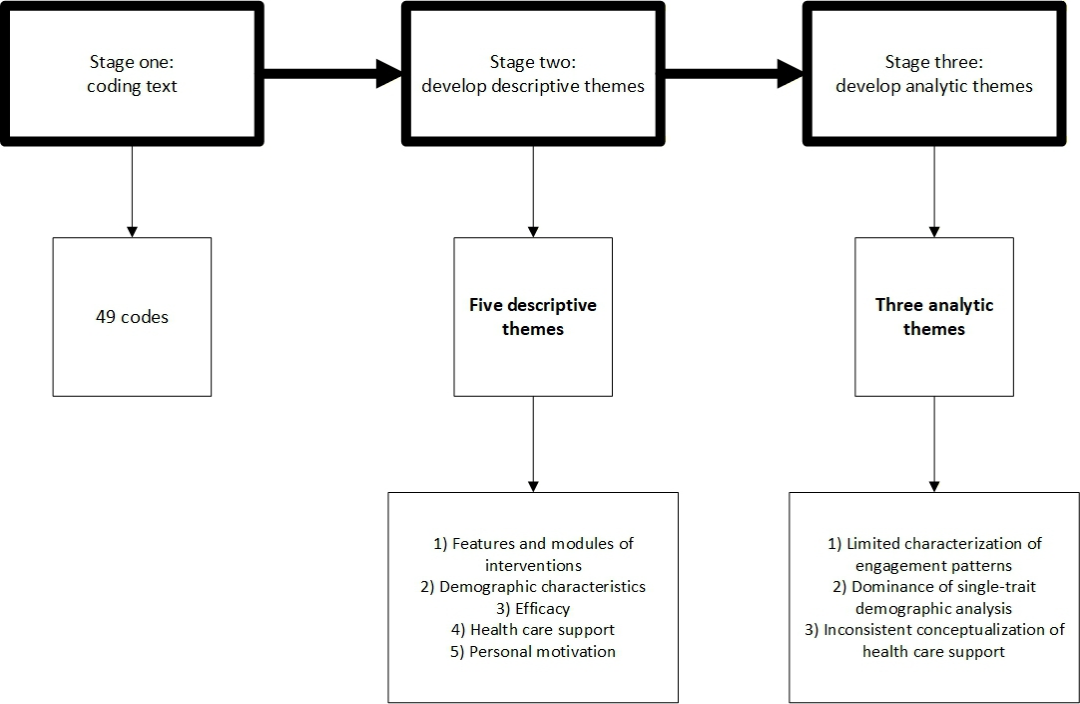
Summary of development of conceptual descriptive themes.

Descriptive thematic analysis yielded 49 codes, 5 descriptive themes, and 3 conceptual descriptive themes (see Figure 5 for an overview). Descriptive themes focused on factors that potentially impacted engagement with digital interventions for asthma and/or COPD; these included (1) features/modules within digital intervention, (2) demographic characteristics, (3) efficacy of the intervention, (4) health care support, and (5) personal motivation. These 5 descriptive themes provided the foundation for 3 overarching conceptual descriptive themes. The following 3 conceptual descriptive themes integrated underlying theoretical frameworks to interpret findings across the included studies.

#### Limited Characterization of Engagement Patterns

Studies that mentioned the potential impact of specific features of an intervention often focused on correlations with outcome measures; for example, “no effects of the peer chat were found on adherence” (to the intervention). Beyond the amount of usage, discussions rarely examined patterns of engagement that might consider changes to usage after an exacerbation or as symptoms increased. The AMUsED framework [35] is identified as a tool that encourages researchers to report and discuss patterns of engagement through the identification of meaningful usage measures. The framework encourages analysts to engage deeply with data and to make informed decisions on how to analyze it.

#### Dominance of Single-Trait Demographic Analysis

Characteristics were often discussed superficially to identify differences between broad characteristics (eg, age, disease severity, and sex). This approach assumes a single characteristic can account for engagement or outcomes, overlooking the “master status identities” described by Hughes [95]. For example, “the fact that females completed more modules than males is in line with gender differences in coping among adolescents.” Intersectionality [96] is a concept that encourages researchers to go beyond single-trait analysis. This is made relevant through Luna’s [97,98] concept of “layered vulnerabilities,” which presents an intersectional approach for applied health research. Additionally, the Health Inequalities Assessment Toolkit (HIAT) [99] is an interactive tool that encourages research processes to develop understanding of health disparities, from design to analysis.

#### Inconsistent Conceptualization of How Health Care Support Affected Engagement

In the limited number of studies where this was discussed, health care support was identified as potentially affecting engagement. However, it was not clear what constituted “encouragement” or “support” from health care professionals (eg, digital literacy, health literacy, and social support). Furthermore, the context of this support (eg, health care setting, remote vs in-person delivery) was rarely specified. Process theories such as that produced by the Medical Research Council might encourage such understanding [100].

## Discussion

### Principal Findings

Developing an understanding of effective engagement could help optimize the design of digital interventions for asthma and COPD. However, this requires being able to identify patterns of engagement through the use of reported measures; for example, comparable usage data and contextually defined outcome measures. This review identified 4 comparable usage measures (frequency of access, in-app time, number of logins, and use of modules) and a range of clinical outcome measures that are commonly reported. Improved reporting of usage data would make quantitative synthesis more feasible. This would require each study to attempt to identify patterns of engagement that lead to effectiveness or to provide datasets with participant-level data. Included studies rarely combined the necessary types of data in analysis that would help identify patterns of engagement. This lack of reporting limited our ability to explore how health inequalities might manifest on digital platforms, despite digital interventions offering a unique opportunity to detect disparities that may not be visible in traditional care pathways. Only 6 studies performed analysis that combined demographic characteristics with outcome or usage data.

Current reporting practices fundamentally limit the ability to understand how engagement drives effectiveness, particularly for underserved populations. Assessing patterns within and between interventions would move us from broad, macro-level claims of efficacy to more precise understandings. More detailed and open reporting of demographic characteristics, particularly those related to recognized health disparities, would encourage analysis of how engagement among subpopulations may impact outcomes [101]. Even where digital infrastructure exists, without comprehensive datasets, developing evidence-based tailored interventions that induce behavior change with multiple strategies will be difficult. Systematic review and meta-analyses of individual participant data provide a method for this type of analysis (eg, Struik et al [102] and Jolliffe et al [103]). This discussion goes into more details followed by a consideration of the limitations.

### Usage Measures

Across the included studies, usage measures were highly heterogeneous, despite the availability of established frameworks such as AMUsED [35] that encourage more systematic reporting. Full-text screening showed that most research on digital interventions for asthma and COPD reported no usage measures at all (n=206, 72.5%). Among the 27 included studies, 4 meaningful and comparable usage metrics were identified, providing a basis for comparison between interventions: 14 studies [13,56,71,73,75,78,80,82,83,85,86,88,89,91] (51.9%) reported only one comparable measure (mean 2.15, SD 0.74), 9 [25,72,74,77,81,84,90,92,94] (33.3%) reported 2, and 4 [76,79,87,93] (14.8%) reported 3. Nine studies [25,56,71,73,75,79,82,91,93](33.3%) reported at least one noncomparable measure (mean 1.6, SD 0.85). Although these represent positive reporting practices, the dominance of simple metrics, typically logins or time spent in the app, provides only a minimal record of interaction and offers a partial view of engagement. Usage data were rarely conceptualized in terms of amount, breadth, or depth, and few studies linked usage patterns to specific intervention components [34,104]. This narrow reporting is striking given that digital interventions routinely collect rich metadata, yet only a small subset is made visible in publications. As a result, opportunities to identify clinically meaningful engagement patterns, such as increased inhaler-related activity preceding symptom deterioration, remain limited, and the development of effective engagement models is constrained by the absence of detailed component-level usage data [36].

From an intersectional perspective, restricted usage reporting also limits the ability to examine whether engagement varies across demographic subgroups or in relation to layered vulnerabilities. Only a small number of studies combined usage with demographic characteristics, and even fewer explored how engagement might differ across intersecting characteristics such as age, sex, ethnicity, SES, or digital literacy. Without richer usage data, these patterns remain obscured. To address this, future research should adopt more comprehensive and theory-informed usage reporting, specifying which components were accessed, how frequently, and at which points in the disease trajectory. Treating usage data as a form of metadata would also allow alignment with established standards such as the findable, accessible, interoperable, and reusable (FAIR) principles [105], supporting more comprehensive reporting practices. This would enable more nuanced analyses of engagement, facilitate the identification of disparities, and strengthen the evidence base needed to tailor digital self-management interventions to diverse patient populations.

### Outcome Measures

Reporting of clinical and self-reported outcome measures was comparatively consistent across the included studies. Outcome measures were grouped into 3 categories (disease-specific self-reported, physiological, and other self-reported), and many studies (n=13, 48.2%) [13,71,72,74,78-81,84,85,87,89,91] reported at least 2 of these categories. Four studies [74,81,84,91] incorporated all 3, providing a multidimensional basis for assessing the potential impacts of digital self-management interventions. Disease-specific self-reported measures (eg, ACQ and C-ACT) were the most frequently used (n=20, 74.1%) [13,72-79,81,82,84,85,87,89-94], reflecting their central role in asthma and COPD research. Physiological outcomes such as FEV₁ were reported less often, but encouragingly, most studies that included physiological measures reported them alongside other outcome types (n=11, 68.8%) [13,71,72,74,78-81,84,85,91], supporting triangulation across domains.

These reporting practices create a strong foundation for comparability across studies, the potential for meta-analyses, and the ability to examine whether specific usage patterns relate to specific outcomes. However, integration of outcomes with demographic and usage data remains limited, meaning that potential disparities in intervention effectiveness are still difficult to identify. For example, it remains unclear how subpopulations may experience differential improvements in symptom control, lung function, or self-management confidence.

Strengthening outcome reporting further will require clearer justification for outcome selection and greater alignment with patient priorities [55,106,107]. Analyzing outcomes alongside demographic and usage data would also enable researchers to identify disparities and understand which populations benefit most. Overall, current reporting practices provide a solid platform from which to build a more comprehensive and equitable evidence base for digital self-management interventions.

### Demographic Characteristics

Reporting of 3 demographic characteristics was common to all studies, including age, disease severity, and sex. On average, studies reported 5.9 demographic characteristics (range 3‐15). However, their use was underwhelming, as Szinay et al [108] reported demographic characteristics of known health inequalities are largely neglected. In our included studies, age was routinely reported, whereas ethnicity, SES, and smoking were often missing. SES, or a proxy of SES, was unreported in half of the included studies (n=14, 51.9%) [25,71,73,75-78,81,83,86-88,92,93]. Similarly, ethnicity was unreported in 82.5% (n=3317) of participants and 70.4% (n=19) of studies; unless studies explicitly focused on ethnicity, participant populations were generally presented as homogeneous. In practice, demographic characteristics were often reported descriptively but rarely used analytically; as such, at best, they imply sample homogeneity. Therefore, little could be accomplished with demographic data.

Improved reporting should be paired with analytic frameworks capable of interrogating how demographic characteristics interact with engagement and outcomes. Greater complexity would encourage discussion that is much more sensitive [97,98]. For example, only 4 studies [72,80,86,88] reported comorbidities despite research evidencing the benefits of managing “treatable traits” that are common across conditions [109]. Existing reporting practices identified in the results, including age, disease severity, and sex, are important, but more characteristics need to be reported and analyzed to develop understanding. A flexible model could be adopted that attempts to identify relevant characteristics at the intervention level. Luna’s [97,98] concept of layered vulnerabilities, like effective engagement, could be applied within specific interventions to support greater comparison between interventions. This would also help identify meaningful demographics, characteristics that go beyond the basics, which might relate to the health care setting, support offered, and the number and type of comorbidities.

Layered vulnerabilities [98] encourage researchers to move away from stereotyping master status identities to generate more nuanced, disease- and intervention-specific taxonomies. The concept contrasts with the idea of “the digital rainbow” [110] and the Prognosis Research Strategy (PROGRESS) framework (place of residence, race/ethnicity/culture/language, occupation, gender/sex, religion, education, SES, and social capital) [111]. Rather than use prescriptive labeling systems, Luna [98] encourages researchers to identify relevant, disease-specific, intersectional disparities as they develop. Luna’s [98] concept could work well with person-centered approaches to statistical analysis [112] as well as participatory and qualitative approaches. Fundamental to achieving this is developing, and reporting a range of diverse and inclusive demographic characteristics.

### Types of Combined Analysis

Many of the included studies did not combine any types of data in analysis (n=17, 62.9%) [13,25,56,71,73,75,78-81,84-87,90,93,94] and no studies combined all 3 types of data in analysis (demographic, outcome, and usage). As others have found [33,113], few studies analyzed usage measures with clinical/physiological or self-reported outcome measures (22.2%). This missing analysis makes it difficult to determine relationships within interventions and to make comparisons between interventions [33]. This was reinforced through the thematic analysis where differences among subpopulations were typically descriptive. Husain et al [106] similarly suggest that research on digital health disparities is typically descriptive and lacking any theoretical basis. Although we use the term “master status identities,” Husain et al [106] used the complementary term, “single-axis analysis.” A combined analysis may offer a more rigorous understanding of effectiveness, within and between interventions, helping to explain differences among subpopulations rather than simply identifying them [34,35]. Nouri et al [55] suggest reporting and responding to different demographic populations is important to increase uptake, sustain engagement, and identify disparities. We highlight analysis by demographic characteristic as crucial to fulfilling the expectations of inclusive research engagement.

### Limitations

This scoping review focused on digital behavior change interventions, and it is likely that the findings are relevant to broader digital health technology (eg, automated sensors or wearable technology). The 2-phase screening approach produced a focused set of usage measures that generated uniformity, overcoming an issue recognized by Nouri et al [55].

The focus on attempting to identify patterns of engagement among heterogeneous populations was problematic. The National Institute for Health and Care Research (NIHR) [114] reports only 60% of RCTs report ethnicity, and they have not yet started to collate data on the reporting of SES. It is important to track recognized disease-specific health inequalities, and digital interventions present a unique opportunity to identify vulnerable characteristics that emerge within datasets. Focusing on “master status identities” potentially magnifies an issue that requires more nuance [96,97].

Finally, this literature review did not develop understanding of organizational or structural factors. Management, resource allocation, and delivery priorities can influence adoption and uptake of digital health interventions and are significant areas to understand. For example, Ramachandran et al [115] suggest barriers and facilitators at the management level impact the adoption of digital interventions for COPD. Scoping reviews on engagement may be a good format to consider if and how management and intersectional factors can be incorporated into research. The results reported here focus on user engagement, and the themes identified overlap with similar research [116,117].

### Implications for Future Research

Existing frameworks such as AMUsED [35] and FAIR [105] have already been highlighted. However, these do not address the identification of health disparities among disease subpopulations. For this, we turned to Luna’s [98] concept of layered vulnerabilities, an intersectional approach that encourages disease- and intervention-specific considerations of demographic disparities. This concept fits well with the [99], an intersectional framework that integrates consideration of health inequalities at all stages of research. However, layered vulnerabilities provide a theoretically informed base that suggests researchers should not wholly rely on existing taxonomies but seek to develop taxonomies through methodology and analysis.

We suggest ways to improve research, with an integrated focus on underserved populations. Rather than a subsidiary research genre, an overarching aim is to bridge the gap between research focused on health inequalities and wider health research. A more concerted effort would encourage greater comprehension of disparities, engagement, and effectiveness for digital health interventions, who is included, how they engage, and who is served. In doing so, evidence-based tailoring options might become apparent. Our recommendations do not rely upon simply increasing “diversity” of samples; indeed, a sample could be homogenous in specific respects (eg, age or ethnicity). We suggest developing meaningful characteristics (eg, delivery site, primary or secondary care, engagement with in-person services, and/or postcode as a marker of environmental exposures). We emphasize two priorities: (1) the identification of meaningful demographics through the expansion of collected characteristics to complicate and complement existing knowledge; and (2) conducting more comprehensive analysis that combines the different types of data. Addressing these priorities in tandem should identify more relevant and sensitive characteristics while encouraging an intersectional analysis.

In summary, a more sensitive intersectional approach to analysis would provide a depth that is currently absent. Disparities should be considered within the epidemiology of the disease and within the matrices of specific digital interventions for a more comprehensive understanding of effectiveness and engagement. Finally, these recommendations are meant to be inclusive rather than prescriptive, not dictating what research should collect, report, or analyze.

### Conclusion

This study advances the field by applying an intersectional lens to digital engagement research, highlighting how current reporting practices limit the ability to identify disparities or tailor interventions. Unlike previous reviews that focus on effectiveness, our analysis maps structural gaps in demographic reporting, outcome selection, and usage analytics. These insights offer actionable recommendations for researchers and developers, supporting more equitable design, evaluation, and implementation of digital self-management interventions in real-world settings.

## Supplementary material

10.2196/73431 Multimedia Appendix 1 Search strategy.

10.2196/73431 Multimedia Appendix 2 Overview of thematic analysis.

10.2196/73431 Multimedia Appendix 3 Guidance for reporting involvement of patients and the public.

10.2196/73431 Multimedia Appendix 4 National Institutes of Health (NIH) quality assessment tool for case-control studies.

10.2196/73431 Checklist 1 PRISMA-S checklist.

10.2196/73431 Checklist 2 PRISMA-ScR checklist.

## Abbreviations

ACQ: Asthma Control Questionnaire
AMUsED: Analyzing and Measuring Usage and Engagement Data
C-ACT: Childhood Asthma Control Test
COPD: chronic obstructive pulmonary disease
FAIR: findable, accessible, interoperable, and reusable
FEV: forced expiratory volume
HIAT: Health Inequalities Assessment Toolkit
ISRCTN: International Standard Randomized Controlled Trial Number
JBI: Joanna Briggs Institute
NIH: National Institutes of Health
NIHR: National Institute for Health and Care Research
PCC: Population, Concept, and Context
PPI: patient and public involvement
PRISMA: Preferred Reporting Items for Systematic Reviews and Meta-Analyses
PRISMA-S: Preferred Reporting Items for Systematic Reviews and Meta-Analyses Literature Search Extension
PRISMA-ScR: Preferred Reporting Items for Systematic Reviews and Meta-Analyses Extension for Scoping Reviews
PROGRESS: Prognosis Research Strategy
SES: socioeconomic status
WHO: World Health Organization
