# Supplementary material for: Engagement and Intersectionality in Digital Self-Management Interventions for Asthma and Chronic Obstructive Pulmonary Disease: Scoping Review
Source: J Med Internet Res. 2026 Jul 23;28:e73431. doi: 10.2196/73431 (PMC13394864; doi:10.2196/73431)
Supplement: Multimedia Appendix 1 [file jmir-v28-e73431-s001.docx]

**Appendix 1: Search Strategy**

The strategy conforms the PRISMA-S guidelines.

**INFORMATION SOURCES AND METHODS**

The following databases were search in February 2023 and last updated in April 2026;

·       AMED (via EBSCO): 57

·       CINAHL (via EBSCO): 890

·       Medline (via EBSCO): 1147

·       PsychInfo (via EBSCO): 381

·       Scopus: 456

The search strategy applied here was not adapted from any existing literature search.

Study registries were not searched.

Online resources and browsing were not used.

Citation searching was not used.

**PEER REVIEW**

The search strategy was peer review by a senior Librarian at University of Southampton before it was finalised – the Librarian was

consulted again at the end to ensure the process was robust and up-to-date.

**MANAGING RECORDS**

The total number of records are cited above and in the flow diagram.

Endnote was used to deduplicate initially, with reviewers verifying these records. Additional duplicates were identified manually in screening.

**JUSTIFICATION FOR LIMITS**

Date limits were used during search updates for convenience.

Expanders were applied to capture equivalent subjects.

There were no limits applied for type of participants, type of research, review process.

No language limits were set.

**DEVELOPMENT OF SEARCH TERMS**

The search terms were developed with all authors. This included a three-month pilot period using free text searches to develop the key terms.

A senior librarian from the University of Southampton was consulted before conducting the search and before performing updates.

MeSH explosion was performed upon advice of the senior librarian.

Text‑word harvesting was not applied.

The first author executed the final search and updates.

Search Strategies were used as planned.

Differences in the number of terms used across databases reflect the indexing structure and search functionality of each platform. In MEDLINE, broad MeSH terms such as *“Pulmonary Disease, Chronic Obstructive+”* automatically explode to include all narrower concepts (e.g., emphysema, chronic bronchitis, COPD overlap syndromes). Adding long synonym lists would not increase retrieval and may reduce precision.

In contrast, databases such as AMED and PsycInfo have less comprehensive indexing or do not support automatic explosion. Therefore, longer free‑text synonym lists were required to ensure sensitive retrieval across varied terminology.

This database‑specific tailoring aligns with PRISMA‑S guidance, which recommends adapting search strings to the indexing behaviour of each database rather than applying identical term lists across platforms.

**SELECTION OF DATABASES**

The selection of databases was agreed with all authors after consultation with the senior librarian.

The selection was based on authority, efficiency, relevance, and the ability to control the search.

Grey literature was not consulted.

**UPDATES**

The search was updated in July 2025 and April 2026 to ensure the most recent records were captured in agreement with the authors.

**AMED Search** (performed May 2026, matched dates to March 2026)

| EBSCO AMED (06MAY2026) | | |  |  |
| --- | --- | --- | --- | --- |
| Print Search History | | |  |  |
|  |  |  |  |  |
| # | Query | Limiters/Expanders | Last Run Via | Results |
| S7 | S5 AND S6 | Limiters - Publication Date: -20260331 | Interface - EBSCOhost Research Databases | 57 |
|  |  | Expanders - Apply equivalent subjects | Search Screen - Advanced Search |  |
|  |  | Search modes - Find all my search terms | Database - AMED - The Allied and Complementary Medicine Database |  |
| S6 | S3 OR S4 | Expanders - Apply equivalent subjects | Interface - EBSCOhost Research Databases | 2,294 |
|  |  | Search modes - Find all my search terms | Search Screen - Advanced Search |  |
|  |  |  | Database - AMED - The Allied and Complementary Medicine Database |  |
| S5 | S1 OR S2 | Expanders - Apply equivalent subjects | Interface - EBSCOhost Research Databases | 4,310 |
|  |  | Search modes - Find all my search terms | Search Screen - Advanced Search |  |
|  |  |  | Database - AMED - The Allied and Complementary Medicine Database |  |
| S4 | "digital health" OR "digital intervention*" OR "mobile health" OR mHealth OR eHealth OR "mobile app*" OR "smartphone app*" OR "web-based" OR "online intervention*" OR telehealth OR telemedicine OR "remote monitoring" | Expanders - Apply equivalent subjects | Interface - EBSCOhost Research Databases | 2,293 |
|  |  | Search modes - Find all my search terms | Search Screen - Advanced Search |  |
|  |  |  | Database - AMED - The Allied and Complementary Medicine Database |  |
| S3 | MH "Digital Technology" OR MH "Artificial Intelligence" OR MH "Computer Applications" OR MH "Digital Video" OR MH "Electronic Communication" OR MH "Mobile Technology" OR MH "Navigation Technology" OR MH "Sensor Technology" OR MH "Streaming Technology" OR MH "Touchscreen Technology" OR MH "Wireless Technologies" | Expanders - Apply equivalent subjects | Interface - EBSCOhost Research Databases | 1 |
|  |  | Search modes - Find all my search terms | Search Screen - Advanced Search |  |
|  |  |  | Database - AMED - The Allied and Complementary Medicine Database |  |
| S2 | COPD OR "chronic obstructive pulmonary disease" OR "chronic obstructive lung disease" OR "chronic airflow limitation" OR "chronic bronchitis" OR emphysema OR "asthma-COPD overlap" OR ACO OR MH "Pulmonary Disease, Chronic Obstructive+" | Expanders - Apply equivalent subjects | Interface - EBSCOhost Research Databases | 2,178 |
|  |  | Search modes - Find all my search terms | Search Screen - Advanced Search |  |
|  |  |  | Database - AMED - The Allied and Complementary Medicine Database |  |
| S1 | asthma* OR "cough-variant asthma" OR "occupational asthma" OR "exercise-induced asthma" OR "aspirin-induced asthma" OR "status asthmaticus" OR "asthma-COPD overlap" OR ACO OR "asthma exacerbation*" OR "severe asthma" OR MH "Asthma+" | Expanders - Apply related words; Apply equivalent subjects | Interface - EBSCOhost Research Databases | 2,297 |
|  |  | Search modes - Find all my search terms | Search Screen - Advanced Search |  |
|  |  |  | Database - AMED - The Allied and Complementary Medicine Database |  |

**CINAHL Search** (April 2026)

| Accessibility Information and Tips | |  |  |  |
| --- | --- | --- | --- | --- |
| Print Search History | |  |  |  |
| # | Query | Limiters/Expanders | Last Run Via | Results |
| S14 | S12 AND S13 | Limiters - Publication Date: -20260331 | Interface - EBSCOhost Research Databases | 890 |
|  |  | Expanders - Apply equivalent subjects | Search Screen - Advanced Search |  |
|  |  | Search modes - Find all my search terms | Database - CINAHL Ultimate |  |
| S13 | S10 OR S11 | Expanders - Apply equivalent subjects | Interface - EBSCOhost Research Databases | 71,376 |
|  |  | Search modes - Find all my search terms | Search Screen - Advanced Search |  |
|  |  |  | Database - CINAHL Ultimate |  |
| S12 | S8 OR S9 | Expanders - Apply equivalent subjects | Interface - EBSCOhost Research Databases | 88,715 |
|  |  | Search modes - Find all my search terms | Search Screen - Advanced Search |  |
|  |  |  | Database - CINAHL Ultimate |  |
| S11 | telemedicine OR Remote Patient Monitoring OR Remote Consultation OR Telepathology OR Distance Counseling OR Telerehabilitation OR Teleradiology OR Mental Health Teletherapy | Expanders - Apply equivalent subjects | Interface - EBSCOhost Research Databases | 42,902 |
|  |  | Search modes - Find all my search terms | Search Screen - Advanced Search |  |
|  |  |  | Database - CINAHL Ultimate |  |
| S10 | MH "Digital Technology" OR MH "Artificial Intelligence" OR MH "Computer Applications" OR MH "Digital Video" OR MH "Electronic Communication" OR MH "Mobile Technology" OR MH "Navigation Technology" OR MH "Sensor Technology" OR MH "Streaming Technology" OR MH "Touchscreen Technology" OR MH "Wireless Technologies" | Expanders - Apply equivalent subjects | Interface - EBSCOhost Research Databases | 30,066 |
|  |  | Search modes - Find all my search terms | Search Screen - Advanced Search |  |
|  |  |  | Database - CINAHL Ultimate |  |
| S9 | copd OR chronic obstructive pulmonary disease OR Asthma-Chronic Obstructive Pulmonary Disease Overlap Syndrome OR Pulmonary Emphysema OR Bronchitis, Chronic | Expanders - Apply equivalent subjects | Interface - EBSCOhost Research Databases | 40,713 |
|  |  | Search modes - Find all my search terms | Search Screen - Advanced Search |  |
|  |  |  | Database - CINAHL Ultimate |  |
| S8 | asthma* OR Cough-Variant Asthma OR Asthma, Occupational OR Asthma-Chronic Obstructive Pulmonary Disease Overlap Syndrome OR Status Asthmaticus OR Asthma, Aspirin-Induced OR Asthma, Exercise-Induced | Expanders - Apply related words; Apply equivalent subjects | Interface - EBSCOhost Research Databases | 53,150 |
|  |  | Search modes - Find all my search terms | Search Screen - Advanced Search |  |
|  |  |  | Database - CINAHL Ultimate |  |
| S7 | S5 AND S6 | Limiters - Publication Date: -20250731 | Interface - EBSCOhost Research Databases | 777 |
|  |  | Expanders - Apply equivalent subjects | Search Screen - Advanced Search |  |
|  |  | Search modes - Find all my search terms | Database - CINAHL Ultimate |  |
| S6 | S3 OR S4 | Expanders - Apply equivalent subjects | Interface - EBSCOhost Research Databases | 49,726 |
|  |  | Search modes - Find all my search terms | Search Screen - Advanced Search |  |
|  |  |  | Database - CINAHL Ultimate |  |
| S5 | S1 OR S2 | Expanders - Apply equivalent subjects | Interface - EBSCOhost Research Databases | 88,715 |
|  |  | Search modes - Find all my search terms | Search Screen - Advanced Search |  |
|  |  |  | Database - CINAHL Ultimate |  |
| S4 | telemedicine OR Remote Patient Monitoring OR Remote Consultation OR Telepathology OR Distance Counseling OR Telerehabilitation OR Teleradiology OR Mental Health Teletherapy | Expanders - Apply equivalent subjects | Interface - EBSCOhost Research Databases | 42,902 |
|  |  | Search modes - Find all my search terms | Search Screen - Advanced Search |  |
|  |  |  | Database - CINAHL Ultimate |  |
| S3 | digital health | Expanders - Apply equivalent subjects | Interface - EBSCOhost Research Databases | 8,493 |
|  |  | Search modes - Find all my search terms | Search Screen - Advanced Search |  |
|  |  |  | Database - CINAHL Ultimate |  |
| S2 | copd OR chronic obstructive pulmonary disease OR Asthma-Chronic Obstructive Pulmonary Disease Overlap Syndrome OR Pulmonary Emphysema OR Bronchitis, Chronic | Expanders - Apply equivalent subjects | Interface - EBSCOhost Research Databases | 40,713 |
|  |  | Search modes - Find all my search terms | Search Screen - Advanced Search |  |
|  |  |  | Database - CINAHL Ultimate |  |
| S1 | asthma* OR Cough-Variant Asthma OR Asthma, Occupational OR Asthma-Chronic Obstructive Pulmonary Disease Overlap Syndrome OR Status Asthmaticus OR Asthma, Aspirin-Induced OR Asthma, Exercise-Induced | Expanders - Apply related words; Apply equivalent subjects | Interface - EBSCOhost Research Databases | 53,150 |
|  |  | Search modes - Find all my search terms | Search Screen - Advanced Search |  |
|  |  |  | Database - CINAHL Ultimate |  |

**Medline Search** (April 2026)

| Accessibility Information and Tips | | |  |  |
| --- | --- | --- | --- | --- |
| Print Search History | | |  |  |
|  |  |  |  |  |
| # | Query | Limiters/Expanders | Last Run Via | Results |
| S11 | S8 AND S9 | Limiters - Publication Date: -20260331 | Interface - EBSCOhost Research Databases | 1,147 |
|  |  | Expanders - Apply equivalent subjects | Search Screen - Advanced Search |  |
|  |  | Search modes - Find all my search terms | Database - MEDLINE |  |
| S10 | S8 AND S9 | Expanders - Apply equivalent subjects | Interface - EBSCOhost Research Databases | Display |
|  |  | Search modes - Find all my search terms | Search Screen - Advanced Search |  |
|  |  |  | Database - MEDLINE |  |
| S9 | S6 OR S7 | Expanders - Apply equivalent subjects | Interface - EBSCOhost Research Databases | Display |
|  |  | Search modes - Find all my search terms | Search Screen - Advanced Search |  |
|  |  |  | Database - MEDLINE |  |
| S8 | S3 OR S4 OR S5 | Expanders - Apply equivalent subjects | Interface - EBSCOhost Research Databases | Display |
|  |  | Search modes - Find all my search terms | Search Screen - Advanced Search |  |
|  |  |  | Database - MEDLINE |  |
| S7 | (MH "Telemedicine+") | Expanders - Apply equivalent subjects | Interface - EBSCOhost Research Databases | Display |
|  |  | Search modes - Find all my search terms | Search Screen - Advanced Search |  |
|  |  |  | Database - MEDLINE |  |
| S6 | (MH "Digital Health") | Expanders - Apply equivalent subjects | Interface - EBSCOhost Research Databases | Display |
|  |  | Search modes - Find all my search terms | Search Screen - Advanced Search |  |
|  |  |  | Database - MEDLINE |  |
| S5 | (MH "Pulmonary Disease, Chronic Obstructive+") | Expanders - Apply equivalent subjects | Interface - EBSCOhost Research Databases | Display |
|  |  | Search modes - Find all my search terms | Search Screen - Advanced Search |  |
|  |  |  | Database - MEDLINE |  |
| S4 | (MM "Asthma+") | Expanders - Apply equivalent subjects | Interface - EBSCOhost Research Databases | Display |
|  |  | Search modes - Find all my search terms | Search Screen - Advanced Search |  |
|  |  |  | Database - MEDLINE |  |
| S3 | asthma* | Expanders - Apply equivalent subjects | Interface - EBSCOhost Research Databases | Display |
|  |  | Search modes - Find all my search terms | Search Screen - Advanced Search |  |
|  |  |  | Database - MEDLINE |  |
| S2 | MH Telemedicine+ | Expanders - Apply equivalent subjects | Interface - EBSCOhost Research Databases | Display |
|  |  | Search modes - Find all my search terms | Search Screen - Advanced Search |  |
|  |  |  | Database - MEDLINE |  |
| S1 | MH Telemedicine+ | Expanders - Apply equivalent subjects | Interface - EBSCOhost Research Databases | Display |
|  |  | Search modes - Find all my search terms | Search Screen - Advanced Search |  |
|  |  |  | Database - MEDLINE |  |

**PsychInfo Search** (performed May 2026 matched dates to March 2026)

| EBSCO Psychinfo | | | | |
| --- | --- | --- | --- | --- |
| Print Search History | | | | |
|  |  |  |  |  |
| # | Query | Limiters/Expanders | Last Run Via | Results |
| S7 | S5 AND S6 | Limiters - Publication Date: -20260331 | Interface - EBSCOhost Research Databases | 381 |
|  |  | Expanders - Apply equivalent subjects | Search Screen - Advanced Search |  |
|  |  | Search modes - Find all my search terms | Database - APA PsycInfo |  |
| S6 | S3 OR S4 | Expanders - Apply equivalent subjects | Interface - EBSCOhost Research Databases | 52,610 |
|  |  | Search modes - Find all my search terms | Search Screen - Advanced Search |  |
|  |  |  | Database - APA PsycInfo |  |
| S5 | S1 OR S2 | Expanders - Apply equivalent subjects | Interface - EBSCOhost Research Databases | 13,360 |
|  |  | Search modes - Find all my search terms | Search Screen - Advanced Search |  |
|  |  |  | Database - APA PsycInfo |  |
| S4 | "digital health" OR "digital intervention*" OR "mobile health" OR mHealth OR eHealth OR "mobile app*" OR "smartphone app*" OR "web-based" OR "online intervention*" OR telehealth OR telemedicine OR "remote monitoring" | Expanders - Apply equivalent subjects | Interface - EBSCOhost Research Databases | 52,586 |
|  |  | Search modes - Find all my search terms | Search Screen - Advanced Search |  |
|  |  |  | Database - APA PsycInfo |  |
| S3 | MH "Digital Technology" OR MH "Artificial Intelligence" OR MH "Computer Applications" OR MH "Digital Video" OR MH "Electronic Communication" OR MH "Mobile Technology" OR MH "Navigation Technology" OR MH "Sensor Technology" OR MH "Streaming Technology" OR MH "Touchscreen Technology" OR MH "Wireless Technologies" | Expanders - Apply equivalent subjects | Interface - EBSCOhost Research Databases | 32 |
|  |  | Search modes - Find all my search terms | Search Screen - Advanced Search |  |
|  |  |  | Database - APA PsycInfo |  |
| S2 | COPD OR "chronic obstructive pulmonary disease" OR "chronic obstructive lung disease" OR "chronic airflow limitation" OR "chronic bronchitis" OR emphysema OR "asthma-COPD overlap" OR ACO OR MH "Pulmonary Disease, Chronic Obstructive+" | Expanders - Apply equivalent subjects | Interface - EBSCOhost Research Databases | 4,235 |
|  |  | Search modes - Find all my search terms | Search Screen - Advanced Search |  |
|  |  |  | Database - APA PsycInfo |  |
| S1 | asthma* OR "cough-variant asthma" OR "occupational asthma" OR "exercise-induced asthma" OR "aspirin-induced asthma" OR "status asthmaticus" OR "asthma-COPD overlap" OR ACO OR "asthma exacerbation*" OR "severe asthma" OR MH "Asthma+" | Expanders - Apply related words; Apply equivalent subjects | Interface - EBSCOhost Research Databases | 10,148 |
|  |  | Search modes - Find all my search terms | Search Screen - Advanced Search |  |
|  |  |  | Database - APA PsycInfo |  |

**Scopus** (14^th^ April 2026)

| Search query |  |  |  |  |  |
| --- | --- | --- | --- | --- | --- |
|  |  |  |  |  |  |
| (  INDEXTERMS("COPD")  OR INDEXTERMS("chronic obstructive pulmonary disease")  OR INDEXTERMS("asthma") ) AND (  INDEXTERMS("Mobile Health")  OR INDEXTERMS("Mobile Applications")  OR INDEXTERMS("Mobile Devices")  OR INDEXTERMS("Digital intervention") ) | | | | | |
|  |  |  |  |  |  |
| Number of records: 456 |  |  |  |  |  |

**https://clinicaltrials.gov/** (315 completed studies – 5^th^ May 2026)

Condition: Asthma OR “Chronic Obstructive Pulmonary Disease” OR COPD

Other terms: digital OR mobile OR smartphone OR app OR web OR telehealth OR telemedicine OR “self‑management”

Study type: Interventional OR Observational

Status: Completed

No date limits were applied. Titles and summaries were screened for relevance to digital self‑management interventions reporting usage, outcome, or demographic data.

[**ISRCTN Registry**](https://www.isrctn.com/) (05MAY2026)

“ISRCTN was searched using paired keyword combinations due to platform constraints. Searches combined respiratory condition terms (‘COPD’, ‘chronic obstructive pulmonary disease’) with digital intervention terms (‘digital’, ‘app’, ‘mobile’, ‘smartphone’, ‘web’, ‘telehealth’, ‘self‑management’). Only completed studies were included because the review required empirical reporting of usage, demographic characteristics, and validated outcome measures. No date limits were applied.”

28 results for COPD AND digital

29 results for COPD AND app

34 results for COPD AND mobile

25 results for COPD AND smartphone

37 results for COPD AND web

8 results for COPD AND telehealth

67 results for COPD AND "self-management"

40 results for "chronic obstructive pulmonary disease" AND digital

21 results for "chronic obstructive pulmonary disease" AND app

32 results for "chronic obstructive pulmonary disease" AND mobile

16 results for "chronic obstructive pulmonary disease" AND smartphone

35 results for "chronic obstructive pulmonary disease" AND web

8 results for "chronic obstructive pulmonary disease" AND telehealth

61 results for "chronic obstructive pulmonary disease" AND "self-management"

54 results for asthma AND digital

42 results for asthma AND app

47 results for asthma AND mobile

30 results for asthma AND smartphone

56 results for asthma AND web

1 results for asthma AND telehealth

51 results for asthma AND "self-management"

*722 total results, 120 unique (96 complete)*

**Reference List of Included Studies**
