# Supplementary material for: Engagement and Intersectionality in Digital Self-Management Interventions for Asthma and Chronic Obstructive Pulmonary Disease: Scoping Review
Source: J Med Internet Res. 2026 Jul 23;28:e73431. doi: 10.2196/73431 (PMC13394864; doi:10.2196/73431)
Supplement: Multimedia Appendix 2 [file jmir-v28-e73431-s002.docx]

**Appendix 2: Overview of Descriptive Thematic Analysis**

This appendix outlines the process used to develop descriptive themes from the included studies. Consistent with the JBI Manual for Evidence Synthesis, the purpose of this analysis was to collate, organise, and map key concepts relevant to the review objectives, rather than to generate new interpretive theory. The approach drew on the first two stages of Thomas and Harden’s (2008) thematic synthesis method: line‑by‑line coding and the development of descriptive themes, adapted for use within a scoping review. Coding was abductive, combining deductive coding (with attention to the review objectives) with inductive coding (identification of concepts that emerged across the dataset).

*Step 1: Data Preparation*

All included studies were imported into NVivo 14. Text after the Results sections (e.g., Discussion, Limitations, Implications, Conclusion) was treated as the analytic dataset, reflecting the aim of exploring how studies described potential gaps in reporting.

*Step 2: Coding of Extracted Text*

The dataset was read in full at least once before coding commenced. Subsequent readings involved semantic, line‑by‑line coding, following Thomas and Harden’s first stage. Codes were iteratively refined through naming and definition to ensure clarity and consistency. Examples of codes and definitions of descriptive themes are provided in Table 1. This process aligns with JBI’s guidance to systematically chart and summarise findings.

*Step 3: Development of Descriptive Themes*

Codes were then grouped into descriptive themes, remaining close to the content of the original text. As recommended by JBI, these themes summarised patterns across studies without moving into interpretive synthesis. Theme names and definitions were refined collaboratively among the authors to enhance transparency and coherence (see Table 1 for an example).

*Step 4: Development of Conceptual Descriptive Themes*

A further layer of descriptive organisation was developed to address the overarching aims of the review. These conceptual descriptive themes remained grounded in the content of the included studies but highlighted conceptual gaps and linked findings to the theoretical framing of the review. This step extended the descriptive analysis without generating new interpretive constructs.

Table 1: Example of Code and Descriptive Themes

| **Descriptive Theme (*definition)*** | **Example Codes (*definition*)** |
| --- | --- |
| Features and Modules of Intervention  *Any code that relates to the perceived impact of specific features within the digital intervention in relation to engagement or outcome.* | Peer chat  *Any text discussing peer-to-peer chat within the intervention*. |
|  | Inhaler Technique  *Any text discussing resources for inhaler technique in the intervention*. |
| Demographic Characteristics  *Any code that relates to perceived differences amongst demographic characteristics in relation to engagement or outcome.* | Age  *Any text discussing the age of users*. |
|  | Sex  *Any text discussing the sex of users*. |
| Efficacy  *Any code that relates to the perceived ability of the intervention to produce the desired result.* | Modules  *Any text that discusses the how a specific module affects engagement or outcome*. |
|  | Time in App  *Any text that discusses how in app time affects engagement or outcome*. |
| Healthcare Support  *Any code that relates to the perceived role of healthcare professionals in affecting patient engagement with the digital intervention.* | Healthcare professional encouragement  *Any text that discusses the potential role of healthcare professionals in affecting engagement with the digital intervention.* |
|  | Integrated Care  *Any text that discusses the perceived difference between usual care pathways and digital care pathways.* |
| Personal Motivation  *Any code that relates to the perceived impact of personal motivation on engagement or outcomes.* | User Priorities  *Any text discussing the perception that user priorities affect engagement or outcome.* |
|  | Subjective experience  *Any text that discusses the perception that user subjective experience of the intervention affects engagement or outcome.* |
