# Supplementary material for: Engagement and Intersectionality in Digital Self-Management Interventions for Asthma and Chronic Obstructive Pulmonary Disease: Scoping Review
Source: J Med Internet Res. 2026 Jul 23;28:e73431. doi: 10.2196/73431 (PMC13394864; doi:10.2196/73431)

[Gripp2 – Short Form](https://www.bmj.com/content/358/bmj.j3453)

PPI Session: Study 1 Systematic review of effective engagement in digital interventions for asthmas and COPD

(19^th^ July 2023)

| ***1: Aim***  *Report the aim of PPI in the study.* |
| --- |
| The aim was to introduce myself, the study, and to facilitate prioritization activities using themes that emerged from the literature review.  This was the first PPI session held for this program of research.  The group has chosen to be referenced as, “The Priory Road Group”.  The session was held after a regular community gathering in the community area of a church.  The group was made up of 11 Afro-Caribbean Elders (9 female, two male), and one Asian (male) person who joined with the intention of seeing what is involved and starting another PPI group for the study. |
| **2: Methods**  *Provide a clear description of the methods used for PPI in the study.* |
| *Matrix scoring* (general): A visual method of analysis in which items are compared according to several criteria. The items are usually listed across the top and the criteria down the side, and a matrix or grid drawn. The boxes are then scored, each item according to each criterion, often using beans, stones, or other counters.  *Preset matrix scoring* - The matrix is made out in advance so that all participants have to do is score the boxes. This is quicker and more comparable, so that overall averages can be worked out. (Both forms of matrix scoring give participants something to put in their reports on a workshop, training or course.)  Activity 1: Preset Matrix Scoring of Themes from the Systematic Review   - The items were preset according to themes that emerged from the literature review. - The criteria was not set, to encourage a more open dialogue (rows). - The group were split into pairs and asked to discuss the matrix, scoring the matrix using seven counters, and record their reasoning.   Activity 2: Present Matrix Scoring of Demographic Characteristics to Report in Research   - The items were preset according to themes that emerged from the literature review (columns). - The criteria was not set, to encourage a more open dialogue (rows). - The group were split into pairs and asked to discuss the matrix, scoring the matrix using seven counters, and record their reasoning.   Chambers, R. (2002) *Participatory Workshops: A Sourcebook of 21 Sets of Ideas and Activities*, London: Routledge |
| **3: Study Results**  *Outcomes—Report the outcomes of PPI in the study, including both positive and negative outcomes.* |
| Engagement features were prioritized as follows   1. clinical support (first), 10 2. how the app works (second), 9 3. who the app is designed for (third), 7 4. research results (fourth), 5 5. Personal motivation (fifth), 1   Demographic characteristics were much more evenly spread – and did not result in a clear prioritization. A Venn diagram drawn by one table could certainly lead to the word ‘intersectionality’ being added to the literature review. |
| **4: Discussion and conclusions**  *Comment critically on the study, reflecting on the things that went well and those that did not, so others can learn from this experience.* |
| The PPI session has encouraged the study to draw attention to and engage in intersectional analyses in the literature review and future empirical work. This was a positive impact. |
| **5: Reflections/critical perspective**  Comment critically on the study, reflecting on the things that went well and those that did not, so others can learn from this experience. |
| The use of matrix scoring was a positive, but could have performed the demographic activity first (since everyone was more familiar with this), and perhaps spend more time explaining the categories of intervention features.  Generally participants expected more explanation of the intervention and purpose however this needs to be balanced with a desire not to influence discussion and outcomes. Despite this, participants engaged with the activities as intended, asking critical questions and objections. |

Activity 1: Preset Matrix Scoring of Themes from the Systematic Review

| ***How the app works*** | ***Who was it designed for?*** | ***What does research show?*** | ***Clinical support*** | **Your Personal Motivation** |
| --- | --- | --- | --- | --- |
| 2 | 3 | 4 | 1 | 5 |
| - Score: 2/3/2/2: 9 - If it gives information about the illness. - Dependent on AI of the app (FAQ and/or customer support). - Easy to access, useability, education – simplicity, availability, user friendly. - Confidence to use, must be simple – easily accessible for those with medical conditions. - Demonstrations and education. | - Score: 2/2/2/1: 7 - Need to know it is helpful for Black people [ie. African diaspora]. - I know its used for people like me – elderly people. - Would give feedback for equal assessment and treatment. - Useful to anyone regardless. | - Score: 3/1/1: 5 - How will we know if it is or not? - Dependent on diagnosis and level of severity. - You want to see how its helping others, friends and general users. - Yes - People should know the benefits. | - Score: 1/2/2/3/2: 10 - We hope that there would be a link. - If I need help, contact is available. - Important with all other … won’t be any good if no access to clinicians. - If one having a crisis definitely to get immediate access would … | - Score: 1: 1 - You want to be able to monitor your own health and attacks. - [need a] Medical diagnosis to be interested. - Motivation also comes with the benefit of keeping fit and healthy. |

Additional comments: Does it work for me, does it help my condition?

Activity 2: Present Matrix Scoring of Demographic Characteristics to Report in Research

| **Age** | **Medical Status** | **Education &**  **Income Status** | **Ethnicity** | **Sex/Gender** | **Smoking History** | **Urban-Rural** |
| --- | --- | --- | --- | --- | --- | --- |
| - Score: 1 - This is especially important for young people. - Very important – elderly are sometimes overlooked. | - Score: 1/1 - Also very important if someone has these symptoms and event if they don’t. [support friends and family] - Healthy or unhealthy. - Existing medical conditions. - [Overlap with age – childhood illnesses can influence]. - Very important. | - Score: 1/1 - Income is very important because it affects peoples wellbeing and state of health. - Income level can determine where and how someone lives (eg pollution, making house safe). | - Score: 2/1 - This makes it inclusive, especially for the first three. - Usually a difference how disease affect ethnicity. - Significance of how cultures impact. - Important. | - Score: 1 - Also very important as illness doesn’t exclude any particular gender. - Usually a difference how disease affect gender. | - Score: 2/1 - Smoking is an important issue. - One of the causes. - Very important, including 2^nd^ hand exposure. - What is smoked and frequency. - Yes important, smoking contributes a lot to illness. | - Score:2/1 - The environment can be crucial to these conditions. - Environment. - Transport. - Impact of emission zone. - It helps where one lives – important. |


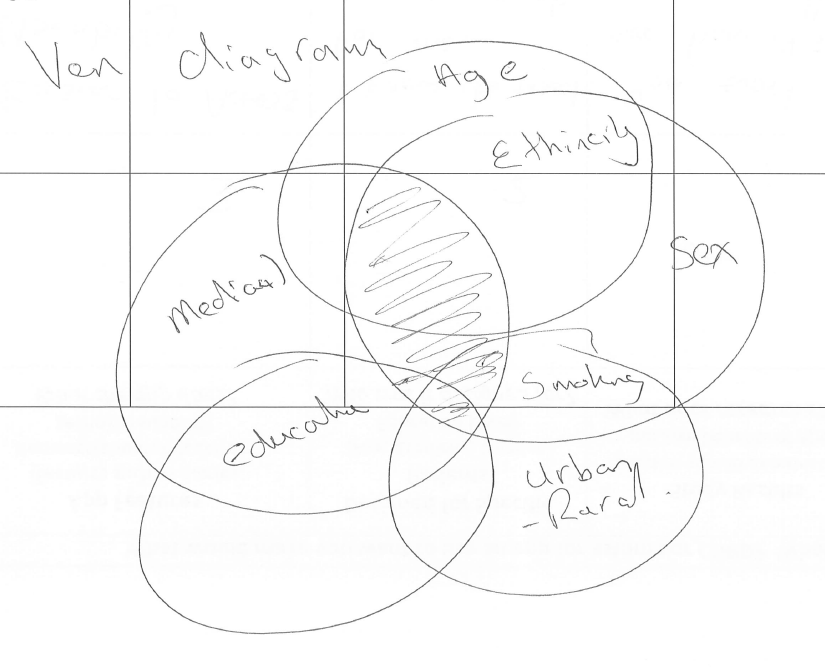

Supplement: Multimedia Appendix 3 [file jmir-v28-e73431-s003.docx]
