# Supplementary material for: Engagement and Intersectionality in Digital Self-Management Interventions for Asthma and Chronic Obstructive Pulmonary Disease: Scoping Review
Source: J Med Internet Res. 2026 Jul 23;28:e73431. doi: 10.2196/73431 (PMC13394864; doi:10.2196/73431)
Supplement: Multimedia Appendix 4 [file jmir-v28-e73431-s004.docx]

| **Criteria** | **Chan et al [71]** | **Gustafson et al [87]** | **Tabak et al [79]** | **Lau et al [73]** | **Newhouse et al [93]** | **Velardo et al [25]** | **Real et al [77]** | **Talboom-Kamp et al [94]** | **Morita et al [82]** | **Kosse et al [76]** | **Boer et al [80]** | **Khusial et al [75]** | **North et al [81]** | **Knox et al [78]** | **Bruzzese et al [92]** | **Greenwell et al [13]** | **Marklund et al [74]** | **Cooper et al [56]** | **Cooper et al [56]** | **Benfante et al [83]** | **Salim et al [85]** | **Glynn et al [86]** | **Genberg et al [88]** | **van der Berg et al [90]** | **Ahmed et al [84]** | **Silverstein et al [89]** | **Silberman et al [91]** |
| --- | --- | --- | --- | --- | --- | --- | --- | --- | --- | --- | --- | --- | --- | --- | --- | --- | --- | --- | --- | --- | --- | --- | --- | --- | --- | --- | --- |
| 1. Was the research question or objective in this paper clearly stated and appropriate? | Yes | Yes | Yes | Yes | Yes | Yes | Yes | Yes | Yes | Yes | Yes | Yes | Yes | Yes | Yes | Yes | Yes | Yes | Yes | Yes | Yes | Yes | Yes | Yes | Yes | Yes | Yes |
| 2. Was the study population clearly specified and defined? | Yes | Yes | Yes | Yes | Yes | Yes | Yes | Yes | Yes | Yes | Yes | Yes | Yes | Yes | Yes | Yes | Yes | Yes | Yes | Yes | Yes | Yes | Yes | Yes | Yes | Yes | Yes |
| 3. Did the authors include a sample size justification? | Yes | Yes | Yes | Yes | Yes | Yes | Yes | Yes | Yes | Yes | Yes | Yes | Yes | Yes | Yes | Yes | Yes | Yes | Yes | Yes | No | Yes | Yes | No | Yes | No | Yes |
| 4. Were controls selected or recruited from the same or similar population that gave rise to the cases (including the same timeframe)? | Yes | Yes | Yes | Yes | Yes | Yes | Yes | Yes | Yes | Yes | Yes | Yes | Yes | Yes | Yes | Yes | Yes | Yes | Yes | Yes | No | Yes | Yes | No | Yes | N/A | Yes |
| 5. Were the definitions, inclusion and exclusion criteria, algorithms or processes used to identify or select cases and controls valid, reliable, and implemented consistently across all study participants? | Yes | Yes | Yes | Yes | Yes | Yes | Yes | Yes | Yes | Yes | Yes | Yes | Yes | Yes | Yes | Yes | Yes | Yes | N/A | N/A | Yes | Yes | Yes | Yes | Yes | Yes | Yes |
| 6. Were the cases clearly defined and differentiated from controls? | Yes | Yes | Yes | Yes | Yes | Yes | Yes | Yes | Yes | Yes | Yes | Yes | Yes | N/A | Yes | Yes | Yes | N/A | N/A | N/A | N/A | Yes | Yes | N/A | Yes | N/A | Yes |
| 7. If less than 100 percent of eligible cases and/or controls were selected for the study, were the cases and/or controls randomly selected from those eligible? | Yes | Yes | Yes | Yes | Yes | Yes | Yes | Yes | Yes | Yes | Yes | Yes | Yes | N/A | Yes | Yes | Yes | N/A | N/A | N/A | N/A | N/A | N/A | N/A | N/A | N/A | N/A |
| 8. Was there use of concurrent controls? | Yes | Yes | Yes | Yes | Yes | Yes | Yes | Yes | Yes | Yes | Yes | Yes | Yes | N/A | Yes | Yes | Yes | N/A | N/A | N/A | N/A | No | N/A | N/A | N/A | N/A | N/A |
| 9. Were the investigators able to confirm that the exposure/risk occurred prior to the development of the condition or event that defined a participant as a case? | Yes | Yes | Yes | Yes | Yes | Yes | Yes | Yes | Yes | Yes | Yes | Yes | Yes | Yes | Yes | Yes | Yes | Yes | Yes | Yes | Yes | Yes | Yes | Yes | Yes | Yes | Yes |
| 10. Were the measures of exposure/risk clearly defined, valid, reliable, and implemented consistently (including the same time period) across all study participants? | Yes | Yes | Yes | Yes | Yes | Yes | Yes | Yes | Yes | Yes | Yes | Yes | Yes | Yes | Yes | Yes | Yes | Yes | Yes | Yes | Yes | Yes | Yes | Yes | Yes | Yes | Yes |
| 11. Were the assessors of exposure/risk blinded to the case or control status of participants? | Yes | Yes | Cannot Determine | Yes | Yes | No | Cannot Determine | Cannot Determine | Cannot Determine | Cannot Determine | No | Cannot Determine | Yes | N/A | Yes | Yes | N/A | N/A | N/A | N/A | N/A | Yes | Yes | N/A | N/A | N/A | N/A |
| 12. Were key potential confounding variables measured and adjusted statistically in the analyses? If matching was used, did the investigators account for matching during study analysis? | Yes | Yes | Yes | Yes | Yes | No | Yes | Yes | Yes | Yes | Yes | Yes | Yes | Partial | Yes | Yes | Yes | Partial | N/A | N/A | N/A | No | No | N/A | Yes | N/A | Yes |

References:

13. Greenwell K, Ainsworth B, Bruton A, et al. Mixed methods process evaluation of my breathing matters, a digital intervention to support self-management of asthma. NPJ Prim Care Respir Med. Jun 4, 2021;31(1):35. [doi: ] [Medline: 34088903]

25. Velardo C, Shah SA, Gibson O, et al. Digital health system for personalised COPD long-term management. BMC Med Inform Decis Mak. Feb 20, 2017;17(1):19. [doi: ] [Medline: 28219430]

56. Cooper R, Giangreco A, Duffy M, et al. Evaluation of myCOPD digital self-management technology in a remote and rural population: real-world feasibility study. JMIR Mhealth Uhealth. Feb 7, 2022;10(2):e30782. [doi: ] [Medline: 35129453]

71. Chan DS, Callahan CW, Hatch-Pigott VB, et al. Internet-based home monitoring and education of children with asthma is comparable to ideal office-based care: results of a 1-year asthma in-home monitoring trial. Pediatrics. Mar 2007;119(3):569-578. [doi: ] [Medline: 17332210]

73. Lau AYS, Arguel A, Dennis S, Liaw ST, Coiera E. “Why Didn’t it Work?” Lessons from a randomized controlled trial of a web-based personally controlled health management system for adults with asthma. J Med Internet Res. Dec 15, 2015;17(12):e283. [doi: ] [Medline: 26678294]

74. Marklund S, Tistad M, Lundell S, et al. Experiences and factors affecting usage of an eHealth tool for self-management among people with chronic obstructive pulmonary disease: qualitative study. J Med Internet Res. Apr 30, 2021;23(4):e25672. [doi: ] [Medline: 33929327]

75. Khusial RJ, Honkoop PJ, Usmani O, et al. Effectiveness of myAirCoach: A mHealth Self-Management System in Asthma. J Allergy Clin Immunol Pract. Jun 2020;8(6):1972-1979. [doi: ] [Medline: 32142961]

76. Kosse RC, Bouvy ML, Belitser SV, de Vries TW, van der Wal PS, Koster ES. Effective engagement of adolescent asthma patients with mobile health–supporting medication adherence. JMIR Mhealth Uhealth. Mar 27, 2019;7(3):e12411. [doi: ] [Medline: 30916664]

77. Real FJ, Beck AF, DeBlasio D, et al. Dose matters: a smartphone application to improve asthma control among patients at an urban pediatric primary care clinic. Games Health J. Oct 2019;8(5):357-365. [doi: ] [Medline: 31157983]

78. Knox L, Gemine R, Rees S, et al. Assessing the uptake, engagement, and safety of a self-management app, COPD.Pal, for chronic obstructive pulmonary disease: a pilot study. Health Technol. May 2021;11(3):557-562. [doi: ]

79. Tabak M, Brusse-Keizer M, van der Valk P, Hermens H, Vollenbroek-Hutten M. A telehealth program for self-management of COPD exacerbations and promotion of an active lifestyle: a pilot randomized controlled trial. Int J Chron Obstruct Pulmon Dis. 2014;9:935-944. [doi: ] [Medline: 25246781]

80. Boer L, Bischoff E, van der Heijden M, et al. A smart mobile health tool versus a paper action plan to support self-management of chronic obstructive pulmonary disease exacerbations: randomized controlled trial. JMIR Mhealth Uhealth. Oct 9, 2019;7(10):e14408. [doi: ] [Medline: 31599729]

81. North M, Bourne S, Green B, et al. A randomised controlled feasibility trial of e-health application supported care vs usual care after exacerbation of COPD: the RESCUE trial. NPJ Digit Med. 2020;3:145. [doi: ] [Medline: 33145441]

82. Morita PP, Yeung MS, Ferrone M, et al. A patient-centered mobile health system that supports asthma self-management (breathe): design, development, and utilization. JMIR Mhealth Uhealth. Jan 28, 2019;7(1):e10956. [doi: ] [Medline: 30688654]

83. Benfante A, Sousa-Pinto B, Pillitteri G, et al. Applicability of the MASK-air app to severe asthma treated with biologic molecules: a pilot study. Int J Mol Sci. Sep 29, 2022;23(19):11470. [doi: ] [Medline: 36232771]

84. Ahmed S, Ernst P, Bartlett SJ, et al. The effectiveness of web-based asthma self-management system, My Asthma Portal (MAP): a pilot randomized controlled trial. J Med Internet Res. Dec 1, 2016;18(12):e313. [doi: ] [Medline: 27908846]

85. Salim H, Cheong AT, Sharif-Ghazali S, et al. A self-management app to improve asthma control in adults with limited health literacy: a mixed-method feasibility study. BMC Med Inform Decis Mak. Sep 27, 2023;23(1):194. [doi: ] [Medline: 37759184]

86. Glynn L, Moloney E, Lane S, et al. A smartphone app self-management program for chronic obstructive pulmonary disease: randomized controlled trial of clinical outcomes. JMIR Mhealth Uhealth. Apr 23, 2025;13:e56318. [doi: ] [Medline: 40267465]

87. Gustafson D, Wise M, Bhattacharya A, et al. The effects of combining web-based eHealth with telephone nurse case management for pediatric asthma control: a randomized controlled trial. J Med Internet Res. Jul 26, 2012;14(4):e101. [doi: ] [Medline: 22835804]

88. Genberg EM, Viitanen HT, Mäkelä MJ, Kautiainen HJ, Kauppi PM. Impact of a digital web-based asthma platform, a real-life study. BMC Pulm Med. May 12, 2023;23(1):165. [doi: ] [Medline: 37173716]

89. Silverstein GD, Styke SC, Kaur S, et al. The relationship between depressive symptoms, eHealth literacy, and asthma outcomes in the context of a mobile health intervention. Psychosom Med. Sep 1, 2023;85(7):605-611. [doi: ] [Medline: 36799736]

90. van den Berg LN, Hallensleben C, Vlug LA, Chavannes NH, Versluis A. The Asthma App as a new way to promote responsible short-acting beta2-agonist use in people with asthma: results of a mixed methods pilot study. JMIR Hum Factors. Apr 4, 2024;11:e54386. [doi: ] [Medline: 38574348]

91. Silberman J, Sarlati S, Harris B, et al. A digital asthma self-management program for adults: randomized clinical trial. JAMA Netw Open. Jul 1, 2025;8(7):e2521438. [doi: ] [Medline: 40674052]

92. Bruzzese JM, George M, Liu J, et al. The development and preliminary impact of CAMP Air: a web-based asthma intervention to improve asthma among adolescents. Patient Educ Couns. Apr 2021;104(4):865-870. [doi: 10.1016/j.pec.2020.09.011] [Medline: 33004234]

93. Newhouse N, Martin A, Jawad S, et al. Randomised feasibility study of a novel experience-based internet intervention to support self-management in chronic asthma. BMJ Open. Dec 28, 2016;6(12):e013401. [doi: ] [Medline: 28031210]

94. Talboom-Kamp E, Holstege MS, Chavannes NH, Kasteleyn MJ. Effects of use of an eHealth platform e-Vita for COPD patients on disease specific quality of life domains. Respir Res. Jul 10, 2019;20(1):146. [doi: ] [Medline: 31291945]
